# Phosphodiesterase 4D Depletion/Inhibition Exerts Anti-Oncogenic Properties in Hepatocellular Carcinoma

**DOI:** 10.3390/cancers13092182

**Published:** 2021-05-01

**Authors:** Federica Ragusa, Nadia Panera, Silvia Cardarelli, Marco Scarsella, Marzia Bianchi, Stefano Biagioni, Mauro Giorgi, Anna Alisi, Mara Massimi

**Affiliations:** 1 Department of Life, Health and Environmental Sciences, University of L’Aquila, 67100 L’Aquila, Italy; federica.ragusa@univaq.it; 2 Research Unit of Molecular Genetics of Complex Traits, Bambino Gesù Children’s Hospital and IRCCS, 00165 Rome, Italy; nadia.panera@opbg.net (N.P.); Marzia.bianchi@opbg.net (M.B.); 3 Department of Biology and Biotechnology Charles Darwin, Sapienza University of Roma, 00185 Rome, Italy; silvia.cardarelli@uniroma1.it (S.C.); stefano.biagioni@uniroma1.it (S.B.); mauro.giorgi@uniroma1.it (M.G.); 4 Flow Cytometry Core Facility, Bambino Gesù Children’s Hospital and IRCCS, 00165 Rome, Italy; marco.scarsella@opbg.net

**Keywords:** PDE, HCC, cell proliferation, apoptosis, IGF2

## Abstract

**Simple Summary:**

Hepatocellular carcinoma (HCC) is one of the leading causes of cancer-related mortality worldwide. Drug resistance is a serious problem in the treatment of HCC. Therefore, it is of high clinical impact to discover targeted therapies that may overcome drug-related resistance and improve the survival of patients affected by HCC. In the present study, we investigated the role of Isoform D of type 4 phosphodiesterase (PDE4D) in HCC development and progression. We found that PDE4D is over-expressed HCCs in vitro and in vivo and the depletion of the gene by silencing or the pharmacological inhibition of protein activity exerted anti-tumorigenic activities.

**Abstract:**

Isoform D of type 4 phosphodiesterase (PDE4D) has recently been associated with several human cancer types with the exception of human hepatocellular carcinoma (HCC). Here we explored the role of PDE4D in HCC. We found that PDE4D gene/protein were over-expressed in different samples of human HCCs compared to normal livers. Accordingly, HCC cells showed higher PDE4D activity than non-tumorigenic cells, accompanied by over-expression of the PDE4D isoform. Silencing of PDE4D gene and pharmacological inhibition of protein activity by the specific inhibitor Gebr-7b reduced cell proliferation and increased apoptosis in HCC cells, with a decreased fraction of cells in S phase and a differential modulation of key regulators of cell cycle and apoptosis. PDE4D silencing/inhibition also affected the gene expression of several cancer-related genes, such as the pro-oncogenic insulin growth factor (IGF2), which is down-regulated. Finally, gene expression data, available in the CancerLivER data base, confirm that PDE4D over-expression in human HCCs correlated with an increased expression of IGF2, suggesting a new possible molecular network that requires further investigations. In conclusion, intracellular depletion/inhibition of PDE4D prevents the growth of HCC cells, displaying anti-oncogenic effects. PDE4D may thus represent a new biomarker for diagnosis and a potential adjuvant target for HCC therapy.

## 1. Introduction

Hepatocellular carcinoma (HCC) represents one of the leading causes of cancer-related mortality worldwide. HCC is a primary liver cancer that in both adults and children develops in the setting of underlying liver diseases, such as non-alcoholic steatohepatitis (NASH), hepatitis B and C and alcohol-related liver disease, but also in the presence of congenital disorders. Thus, the tumour mechanisms of onset and progression are diverse and contribute to the complexity of studying and treating HCC [1,2].

Curative treatments to provide long-term survival for HCC patients in the early stages include surgical resection, radiofrequency ablation, or liver transplantation. Systemic chemotherapy and targeted drugs remain the approaches of choice for patients with advanced HCC [3]. Currently, systemic therapy for HCC mainly consists of multikinase inhibitors and/or tyrosine kinase receptor inhibitors including sorafenib, regorafenib and lenvatinib [4,5]. However, the survival benefit of these drugs for patients with HCC is unsatisfactory due to the development of drug resistance [6]. Therefore, the discovery of adjuvant targeted therapies that may overcome drug-related resistance and improve the survival of patients affected by HCC remains challenging.

Recently, modulation of cyclic adenosine monophosphate (cAMP) has been shown to be of great interest in the control of cell proliferation in different tumour cell lines [7]. Most tumour cells have lower levels of intracellular cAMP when compared to healthy cells and an inverse relationship between cAMP levels and degree of malignancy has also been described. In these tumour cells, stimulation of cAMP synthesis and/or inhibition of cAMP degradation could lead to increased levels of this nucleotide and thus to a possible slowing down of cell proliferation. The duration of the cAMP signal is regulated by the activity of both the adenylyl cyclase enzyme, which promotes cAMP synthesis, and the phosphodiesterase (PDE) enzyme, which promotes the hydrolysis of cAMP to 5′AMP [8].

PDEs are up-regulated in most human tumours and thus represent important targets for the pharmacological control of cAMP and of numerous intracellular cAMP-dependent effectors [9]. In this context, the use of PDE inhibitors, as agents able to increase cAMP levels, may represent a novel therapeutic approach for several types of cancers, including HCC [10,11]. PDEs are classified in 11 families (PDE1-PDE11). Some of them, including PDE1, 2, 3, 10, and 11, control the intracellular levels of cAMP and cyclic guanosine monophosphate (cGMP); while PDE4, 7, and 8 are specific for cAMP degradation [12]. Noteworthy, PDE4 has been described as a regulator of cAMP-hydrolysing activity in several human cancer cells [11]. PDE4 is encoded by four genes, which gives rise to four different isoforms (A, B, C and D). PDE4 isoforms A, B and D are abundantly expressed in liver, and they have been proposed as possible targets in the treatment of alcoholic liver disease [13] and cholestatic liver injury/fibrosis [14]. Massimi et al. [15] observed that HCC cells displayed a cAMP/cGMP hydrolysis activity ratio higher than non-tumorigenic hepatocytes due to an increase of cAMP hydrolysis, mostly achieved by PDE4. The same authors also demonstrated that treatment with PDE4 inhibitors affected the cell cycle and survival. Moreover, recently Peng et al. [16] reported that PDE4A is aberrantly over-expressed in HCC tissues, where it predicts the worse post-surgical outcome, which could be dependent on the induction of an epithelial to mesenchymal transition. However, data on the role of isoform D of PDE4 (PDE4D) in HCC still needs to be elucidated.

Hence, in the present study, firstly we examined the expression of PDE4D in HCC tissues and adjacent liver tissues, and their possible correlation with tumour characteristics. Next, we evaluated the anti-oncogenic properties of PDE4D depletion or inhibition in different HCC cell lines.

## 2. Materials and Methods

### 2.1. Tissue Microarray Construction and Immunofluorescence

A customized tissue microarray (TMA) containing 5 paraffin-embedded normal liver tissues and 16 paraffin-embedded HCCs (age range from 3 to 68 years), was purchased from BioChain (Newark, CA, USA), and used for immunofluorescence staining. The company also provided the grade of tumour specimen based on the 4-scale Edmondson and Steiner system but information about tumour aetiology was not available.

Antigen retrieval was performed with EDTA (pH 8) (Dako, Glostrup, Denmark). The primary antibody against PDE4D (AB83940, dilution 1:100; Immunological Sciences, Rome, Italy) were added and incubated overnight at +4 °C. The primary antibody was revealed with a goat anti-rabbit Alexa Fluor^®^ 488 secondary antibody (Invitrogen, Thermo Fisher Scientific Inc., Waltham, MA, USA). Nuclei were counterstained with 4′,6-diamidino-2-phenylindole (DAPI) for 5 min after extensive washing; sections were mounted with PBS/glycerol (1:1) and covered with a coverslip. Confocal microscopy imaging was performed on a Fluoview FV1000 confocal microscope (Olympus, Tokyo, Japan ) equipped with FV10-ASW version 2.0 software, using 20×, 40× and 60× objectives.

Fluorochrome unmixing was performed by acquisition of automated-sequential collection of multi-channel images, to reduce spectral crosstalk between channels. Sections were acquired with a format of 1024 × 1024 pixels, a sampling speed of 40 µs/pixel, and 12 bits/pixel images. For imaging analysis, the average intensity of fluorescence (arbitrary units) was calculated from ten measurements of specific regions of interest in at least three randomly selected digital images using Image J software. Primary and secondary antibodies are reported in Table S1.

Cell immunofluorescence was performed on HCC cells seeded at a density of 20,000 cells/well in 4-well chamber slides and fixed in cold methanol/acetone (3:1). The slides were incubated with the primary antibody against IGF2 (AB9574, dilution 1:200; Abcam Inc., Cambridge, MA, USA). Nuclei were counterstained with DAPI. Detection of the primary antibody was performed using goat anti-rabbit Alexa Fluor^®^ 488 antibody. The images were captured and analyzed using an Eclipse E600 epi-fluorescence microscope (Nikon, Tokyo, Japan) equipped with NIS-Elements BR 3 0 Software, using a 40× objective.

### 2.2. Gene Expression Profiling Interactive Analysis (GEPIA)

Data of PDE4D gene expression in HCCs were obtained using gene expression profiling interactive analysis (GEPIA). The web server was used to analyse the RNA sequencing expression data of HCCs compared with 50 normal liver using a standard processing pipeline [17].

### 2.3. Cell Lines, Cultures and Drug Treatments

HepG2 cells were purchased from American Type Culture Collection (ATCC, Manassas, VA, USA), Hep3B cells were from Sigma-Aldrich (St. Louis, MO, USA), Huh7 cells were from the JCRB collection. HCC cell lines (HepG2 from a 15-year-old Caucasian male; Hep3B from a 8-year-old black male from the United States, and Huh7 from a 75-year-old Japanese male) were grown in Dulbecco’s modified Eagle’s medium-low glucose (Euroclone, Milan, Italy) supplemented with 10% foetal bovine serum, 2 mM L-glutamine, 100 μg/mL streptomycin and 100 U/mL penicillin, at 37 °C and 5% CO_2_ in a humidified atmosphere. Cells were all seeded at a concentration of 104 per cm2 and treated for the indicated times with the chosen concentrations of inhibitors or with vehicle alone. Before treatments, cells were starved for 24 h in the same medium containing lower serum concentration (0.5%) to allow synchronization. All cell lines were intermittently tested for the presence of mycoplasma.

Differentiated human HepaRG cells (Gibco, Thermo Fisher Scientific Inc.) were plated at a concentration of 100 × 104/cm^2^ in Williams’ Medium E, supplemented with 10% fetal bovine serum, 1% GlutaMAX™-1, 100 μg/mL streptomycin, 100 U/mL penicillin and 5 μg/mL insulin, at 37 °C in a 5% (*v*/*v*) CO_2_ humidified atmosphere, and used after 48 h. For the treatment with PDE4D inhibitor, (*E*)-3-(cyclopentyloxy)-4-methoxy-benzaldehyde, O-2-(2,6-dimethylmorpholino)-2-oxoethyl oxime (Gebr-7b, Calbiochem^®^, Merk Millipore, Darmstadt, Germany) was dissolved in dimethyl sulfoxide (DMSO, Sigma-Aldrich) and stored as a stock solution in small aliquots at −20 °C until time of use. The final DMSO concentration was kept constant in each experiment (less than 0.1% *v*/*v*).

### 2.4. Quantitative Real-Time PCR (qRT-PCR)

Total RNA extraction was performed using Trizol (Invitrogen, Thermo Fisher Scientific Inc.) according to the manufacturer’s protocol and inspected by agarose gel electrophoresis. cDNA was synthesized with the High Capacity cDNA Reverse Transcription Kits (Applied Biosystems, Thermo Fisher Scientific Inc.), according to the kit’s instructions. Quantitative Real-Time (qRT-PCR) amplification, detection and analysis was performed with the ABI Prism 7900HT Fast Real-Time PCR System (Applied Biosystems, Thermo Fisher Scientific Inc.) using SensiFast PCR Master Mix (2×) No AmpErase^®^ UNG (Applied Biosystems, Thermo Fisher Scientific Inc.). The samples were normalized according to the glyceraldehyde-3-phosphate dehydrogenase (GAPDH) and snRNA mRNA levels. Based on the ΔΔCt method, relative amounts of mRNA were expressed as fold changes versus controls. TaqMan gene assays for GAPDH (Hs99999905_m1), PDE4D (Hs01579625_m1), and insulin growth factor 2 (IGF2) (Hs01005963_m1) were purchased from Applied Biosystems (Thermo Fisher Scientific Inc.,).

For the analysis of PDE4A, PDE4B and PDE4C mRNA, the cDNAs containing 2x SYBR Green PCR Master Mix (Applied Biosystems, Thermo Fisher Scientific Inc.) were used for the qRT-PCR. All samples were run in triplicate. The cDNAs were amplified in QuantStudio™ 12K Flex Real-Time PCR System (Applied Biosystems, Thermo Fisher Scientific Inc.). All primer were purchased from Sigma-Aldrich. Primers list: PDE4A (NM_006202): 5′-GCTGAAGACCTCATCGTAAC-3′ (forward), 5′-ATTCTGTTTGTCCAGGAATG-3′ (reverse); PDE4B (NM_002600): 5′-ATTCTGTTTGTCCAGGAAT-3 (forward)’, 5′-ATGCTGGTGTAGAAAGGAGA-3′ (reverse); PDE4C (NM_000923): 5′-AGAGTGGT ACCAGAGCAAGA-3′ (forward), 5′-TGGGAGCCACCTATAACTAA-3′ (reverse), and normalized to the level of GAPDH 5′-TCCAAAATCAAGTGGGGCGA-3′ (forward), 5′-TGATGACCCTTTTGGCTCCC-3′ (reverse) using the comparative ΔCt method.

### 2.5. Western Blotting

Cells were collected and total protein extraction was performed with RIPA lysis buffer (Cell Signaling Technology, Inc, Danvers, MA, USA) containing 1× protease and phosphatase inhibitors cocktail. The homogenates were then centrifuged at 14,000 *g* at +4° for 10 min and the resulting supernatant was taken as a protein sample. Whole cell extracts were quantified using the BCA™ Protein Assay (Thermo Scientific, Thermo Fisher Scientific Inc.). Samples were then diluted in the sample buffer (200 mM Tris-HCl (pH 6.8), 40% glycerol, 20% β-mercaptoethanol, 4% sodium dodecyl sulphate, and bromophenol blue) and resolved by SDS-PAGE, then transferred and immobilized onto nitrocellulose membranes (GE Healthcare Life Sciences, Munich, Germany). The membranes were blocked using 5% non-fat dry milk for 30 min and incubated with the appropriate primary and secondary antibodies.

GAPDH or β-actin were used as internal controls for equal protein loading. Western blotting was performed in duplicate for at least three independent experiments. Protein expression was quantified by densitometric analysis using ImageJ v3.91 software. Primary and secondary antibodies are listed in Table S1. All uncropped Western blot figures can be found in Figure S8.

### 2.6. Cell Homogenization, PDE Activity and Cyclic AMP Level Assay

Cells were collected and homogenized in a 20 mM Tris-HCl buffer, pH 7.2, with 0.2 mM EGTA, 5 mM MgCl_2_, 1 mM phenylmethylsulfonyl fluoride, 5 mM 2-mercaptoethanol, 2% (*v*/*v*) antiprotease cocktail (Sigma-Aldrich) and 0.1% Triton X-100 using a mini-glass homogenizer (15 strokes, 4 °C). All procedures were performed at 4 °C. The homogenate was centrifuged at 14,000 *g* for 30 min at 4 °C and the resulting pellets were resuspended in the homogenization buffer and centrifuged again at 14,000× *g* for 30 min. The first and the second supernatants were then pooled and used for further analysis.

PDE activity was measured with the method described by Thompson and Appleman [18] at 30 °C in 60 mM Hepes pH 7.2, 0.1 mM EGTA, 5 mM MgCl_2_, 0.5 mg/mL bovine serum albumin (BSA), and 30 mg/mL soybean trypsin inhibitor, in a final volume of 0.15 mL. The reaction was started by adding tritiated substrates at a final concentration of 1 µM [3H] cAMP and stopped by adding 0.1 N HCl. After neutralization with 0.1 N NaOH in 0.1 M Tris–HCl, pH 8.0, 2 mg/mL 5′-nucleotidase (snake venom from *Crotalus atrox*; Sigma-Aldrich) in 0.1 M Tris–HCl, pH 8.0, were added. Samples were gently mixed and incubated for 30 min to allow complete conversion of 5′-nucleotide to its corresponding nucleoside. Unhydrolyzed cyclic nucleotide and the corresponding nucleoside were separated by DEAE-Sephadex A-25 columns. The eluate was mixed with ULTIMA GOLD scintillation liquid (PerkinElmer, Waltham, MA, USA,) and counted on a Tri-Carb 2100TR Liquid Scintillation Counter (2000CA; Packard Instruments, Merideny, CT, USA). To evaluated PDE4D specific activity from total cAMP PDE activity, the PDE4D inhibitor Gebr-7b was added to the reaction mix at 30 µM final concentration. PDE4D activity was calculated as the difference between the total hydrolytic activity and the residual hydrolytic activity assayed in the presence of the specific inhibitor.

To measure cyclic AMP levels, seeded cells were treated with 5 µM Gebr-7b for 1 h, washed in PBS and rapidly homogenized in 0.1M HCl. The homogenates were centrifuged at 10,000× *g* and the supernatants were acetylated using a Direct EIA kit (Enzo Life Sciences Inc., Farmingdale, NY, USA), according to the manufacturer’s instructions.

### 2.7. Small Interference RNA (siRNA) Transfection

HCC cells were transfected with various siRNAs directed against PDE4D (3 unique 27mer siRNA duplexes—2 nmol each, Locus ID 5144) and with a control siRNA (Trilencer-27 Universal Scrambled Negative Control siRNA Duplex—2 nmol), all purchased from OriGene Technologies, Inc. (Rockville, MD, USA). The siRNA-27 kit contains three Dicer-Substrate 27-mer duplexes targeting a specific gene that are selected from a predesigned set of duplexes from the RefSeq collection of human Genbank, therefore the sequences are tested by OriGene that is proprietary of the kit. Briefly, a 20 μM stock solution of siRNAs was prepared and then the siRNAs were tested at two different concentrations (10 nM and 20 nM). Sequences of siRNA were: GGAATCAACTAGTTAATTGCAAGGT (SR303409A); GCAATTAACTGATTTGTAGTG ATTC (SR303409B); CCACTAAGATAAACCAAATGTCCTT (SR303409C).

The cells were seeded at a density of 5 × 10^5^ cells/well into 6-well dishes and cultured overnight at 37 °C with 5% CO_2_ until the cells reached 70% confluency. Transfections for all relevant data were performed using 20 nM siRNAs and Invitrogen Lipofectamine 2000 reagent and (Thermo Fisher Scientific Inc.), according to the manufacturer’s protocol.

### 2.8. DELFIA Cell Proliferation Assay

Cells were plated in a 96 microplate well and allowed to grow. The Dissociation-Enhanced Lanthanide Fluorescent Immunoassay (DELFIA) Cell Proliferation Assay was performed following the manufacturer’s instructions (PerkinElmer). Specifically, cells were incubated with bromodeoxyuridine (BrdU) for 3 h at 37 °C. The europium-labelled antibody was used to detect incorporated BrdU following the DELFIA Cell Proliferation Assay protocol. The dissociation of europium ions from the anti-BrdU antibody and the formation of their fluorescent chelates were obtained by DELFIA inducer reagent. The fluorescence, which is proportional to DNA synthesis, was measured by time-resolved fluorometer 2100 EnvisionTM Multilabel Reader (PerkinElmer).

### 2.9. Cell Cycle and Apoptosis

Cell cycle analysis was performed by FACS at 48 h post-starvation serum replacement by flow cytometry using PI staining (Sigma-Aldrich). Briefly, cells were collected by trypsinization, washed with PBS, then fixed in a solution of a cold 4:1 methanol/acetone solution. Cells were first incubated with RNase A at +37 °C then stained with a solution containing 100 μg/mL PI, at +37 °C for 20 min. The stained nuclei were analysed for DNA-PI fluorescence using a Becton Dickinson FACSCanto II flow cytometer (Becton-Dickinson, Milan, Italy). Resulting DNA distributions were analysed for the proportion of cells in G0/G1, S phase, and G2/M phases of the cell cycle by DiVa Software, version 6.3 (Becton-Dickinson).

Apoptosis was assessed by Annexin V staining at 24 h from post-starvation serum replacement. Briefly, cells were washed in PBS and re-suspended in Annexin Binding Buffer (10 mmol/L HEPES pH 7.4, 140 mmol/L NaCl, and 2.5 mmol/L CaCl). Cells were then stained with 0.5 mg/mL Annexin V-FITC (Becton-Dickinson) for 15 min before the analysis. Acquisition and analysis were carried out on a Becton Dickinson FACSCanto II flow cytometer, using DiVa Software, version 6.3.

### 2.10. Open Array

A pre-designed TaqMan OpenArray Human Cancer Panel (Life Technologies) was used to assess the effect of PDE4 depletion in HepG2 cells on a signature panel of 624 well-defined genes validated for the characterization of cancers [19], plus 24 endogenous control genes. cDNAs were loaded onto the Open Array platform and run as recommended by the manufacturer on the QuantStudio 12K Flex Real-Time PCR system (Applied Biosystems, Thermo Fisher Scientific Inc.). Raw data were extracted from the QuantStudio 12 K Flex software and analysed using a software in cloud provided as a service by Life Technologies. This software converts CT values to relative quantity (RQ) expression levels for further analysis using a standard delta–delta transformation and R for statistics. RQ minimum and maximum values were calculated with a confidence level of 95%, using Benjamini-Hochberg false discovery rate to adjust *p* values. Maximum allowed Ct included in calculations was 35 and Cq confidence > 0.8. Multivariate Student’s t-test was applied and values of *p* < 0.05 were considered statistically significant.

### 2.11. Statistics

The data were presented as the means ± SD. Comparisons were made between the means from at least two independent experiments repeated in triplicate. Statistical differences were analysed by using Student t test or ANOVA. *p* values < 0.05 were considered statistically significant; * *p* < 0.05, ** *p* < 0.01, *** *p* < 0.001.

## 3. Results

### 3.1. PED4D Protein/Gene Are Over-Expressed in HCC Tissues and Cell Lines

**Figure 1:**
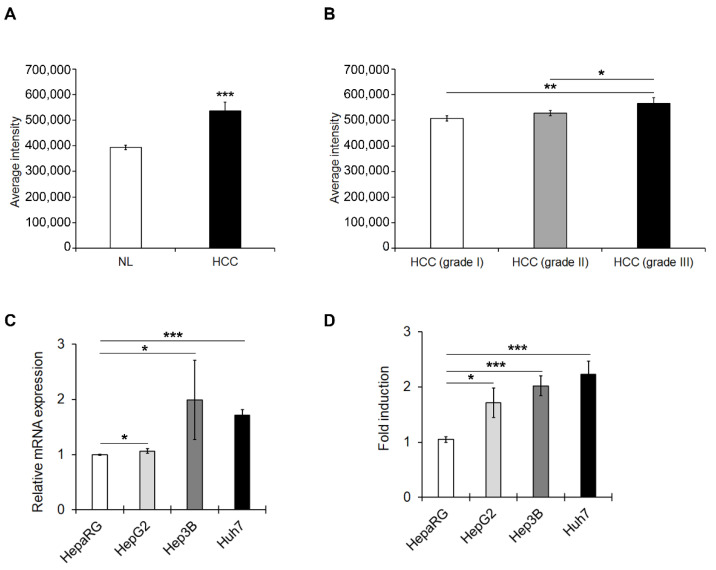
PDE4D protein/gene expression levels in HCC tissues and HCC cell lines. (**A**) Expression levels of PDE4D measured as average intensity of fluorescence in HCC tissues (HCC) compared to normal liver samples (NL). (**B**) Expression levels of PDE4D measured as average intensity of fluorescence in HCC samples stratified by tumour histological grading. (**C**) qRT-PCR analysis of mRNA levels of PDE4D in HCC cells compared to well-differentiated HepaRG cells. GAPDH was used as a housekeeping gene. (**D**) The relative expression of PDE4D measured by semi-quantitative analysis of the Western blots in HCC cells normalized to GAPDH and reported as fold-changes compared to well-differentiated HepaRG cells. Data are the mean ± standard deviation (SD) of three independent experiments. Student t or Anova test. * *p* < 0.05; ** *p* < 0.01; *** *p* < 0.001.

The expression of PDE4D protein was first examined by immunofluorescence of commercially available TMA that contained 5 normal liver tissues (NL) and 16 HCC samples from adults and children. Quantitative analysis of the imaging revealed that the expression levels of PDE4D protein were up-regulated in HCC compared to NL (Figure 1A and Figure S1A). Moreover, the expression of PDE4D in HCC progressively increased with severity of the disease in terms of tumour histological grade (Figure 1B).

Analysis of PDE4D gene expression was next performed by the interactive tools available on the GEPIA website (http://gepia2.cancer-pku.cn/#analysis, accessed on 22 February 2021). The analysis of 50 normal liver samples and 126 HCC samples highlighted that the PDE4D gene was significantly over-expressed in HCCs compared to normal livers (Figure S1B). Interestingly, when we explored with GEPIA the connections between PDE4D gene expression and overall survival in the above-mentioned samples, the Kaplan-Meier curve revealed that HCC patients with higher levels of PDE4D expression showed a worse prognosis than those with a lower level of PDE4D expression, but the difference was not statistically significant (Figure S1C).

Accordingly, PDE4D mRNA and protein expression were detected in 3 human HCC cell lines (HepG2, Huh7, and Hep3B) with different degrees of malignancy and compared to well-differentiated HepaRG that resemble much more normal human hepatocytes. There was a significant up-regulation in the PDE4D mRNA (Figure 1C) and protein expression (Figure 1D and Figure S2) in the three cell lines, in Huh7 and Hep3B in particular, compared to HepaRG cells.

**Figure 2:**
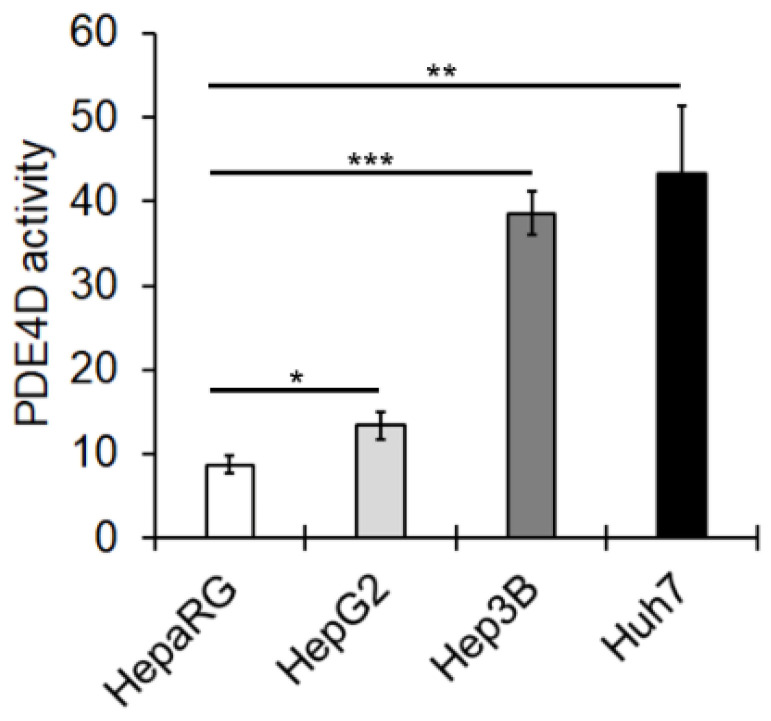
PDE4D enzymatic activity in HCC cells. PDE4D activity was evaluated as described in the Materials and Methods section and expressed as pmol of cAMP hydrolyzed/min/mg protein. Data are the mean ± SD of three independent experiments. Anova test. * *p* < 0.05; ** *p* < 0.01; *** *p* < 0.001.

In order to verify whether up-regulation of PDE4D mRNA and protein was associated with increased protein activity, we tested cAMP hydrolysing activity levels in the mentioned HCC cell lines. The results showed that PDE4D activity increased in HCC cell lines, particularly in those with a more aggressive phenotype (Huh7 and Hep3B) (Figure 2).

### 3.2. Silencing of PDE4D Affects cell Proliferation, Cell Cycle and Apoptosis of HCC Cells

HepG2, Huh7 and Hep3B transiently transfected with scrambled (Scr-siRNA) or PDE4D-specific siRNAs (PDE4D-siRNA) were established. As shown in Figure S3A–C, 20 nM siRNAs caused, 48 h after transfection, at least a 50% reduction of PDE4D mRNA expression, but no significant changes in the expression PDE4A, PDE4B and PDE4C mRNA (Figure S3D–F). The silencing-dependent decrease of the PDE4D gene was reflected in a dramatic reduction of protein expression (Figure S4A–C) and activity (Figure S4D–F).

**Figure 3:**
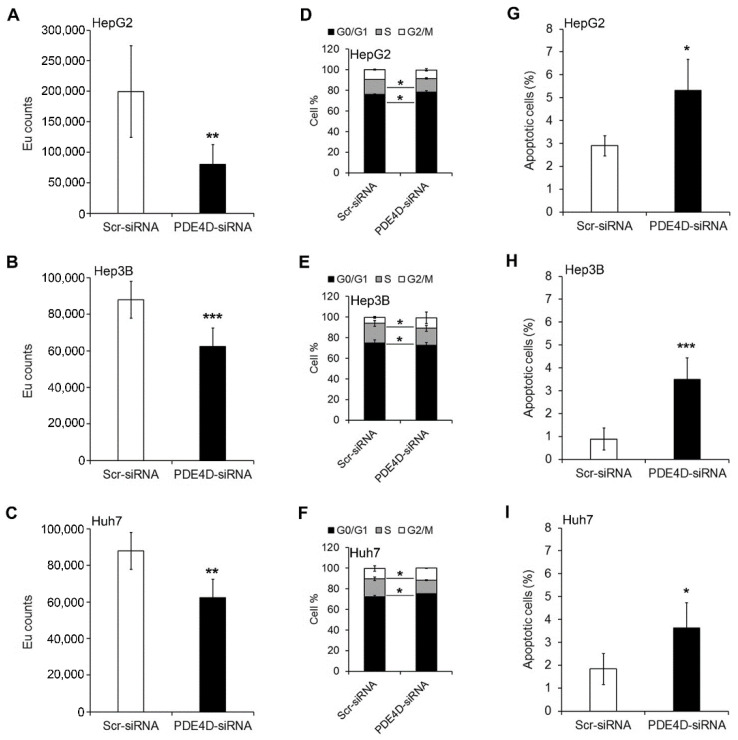
The effect of PDE4D gene silencing on proliferation, cell cycle, and apoptosis in HCC cells. Cell proliferation assay performed by DELFIA assay in HepG2 (**A**), Hep3B (**B**), and Huh7 (**C**) cells. Value expressed in EU counts. Cell cycle profiles measured by propidium iodide staining and flow cytometry analysis in HepG2 (**D**), Hep3B (**E**), and Huh7 (**F**) cells. Flow cytometry evaluation of apoptosis using Annexin V-FITC/PI staining in HepG2 (**G**), Hep3B (**H**), and Huh7 (**I**) cells. Data are the mean ± SD of three independent experiments. Student t test. * *p* < 0.05; ** *p* < 0.01; *** *p* < 0.001 versus Scr-siRNA.

In order to evaluate the effects of PDE4D silencing on HCC proliferation, a BrdU incorporation assay was performed. As shown in Figure 3A–C, cell proliferation was significantly reduced after 48 h in PDE4D-siRNA HCC compared to Scr-siRNA HCC cells. Accordingly, the percentage of PDE4D-siRNA cells in S phase was significantly lower than that of Scr-siRNA cells with a concomitant increase of cells in G0/G1 phase (Figure 3D–F and Table S3). The reduction of cell proliferation was also accompanied by an induction of apoptosis. Indeed, as shown in Figure 3G–I, Annexin V analysis demonstrated that the percentage of apoptotic cells was significantly higher in the PDE4D-siRNA cells than in the Scr-siRNA HCC cells.

**Figure 4:**
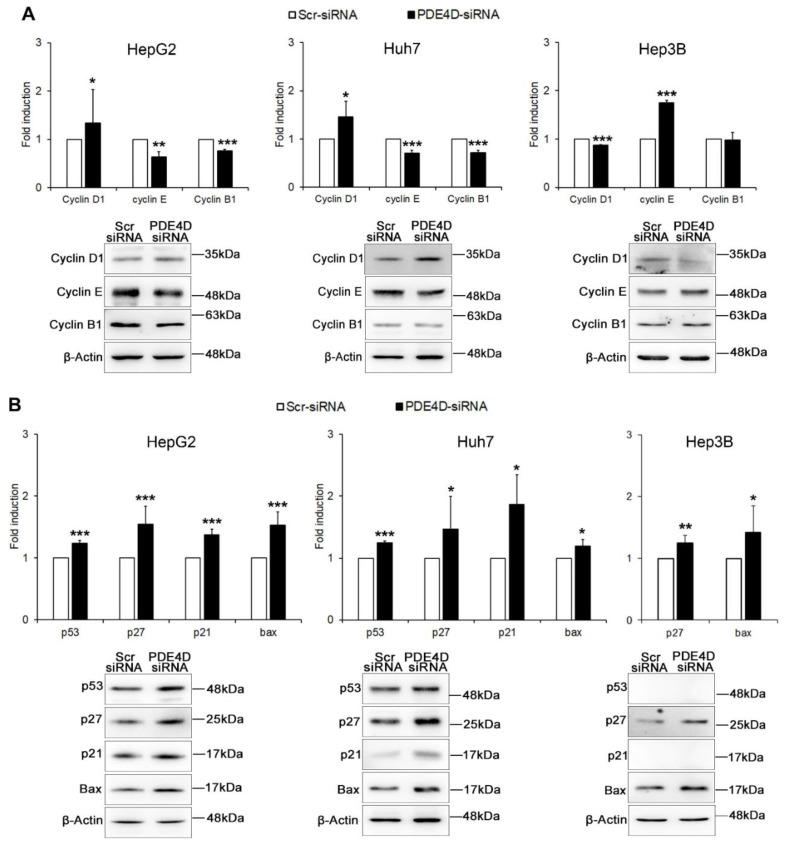
The effect of PDE4D gene silencing on cell cycle regulators and apoptotic proteins. Western blot analysis in HepG2, Huh7 and Hep3B cells 48 h after transient silencing. The graphs represent densitometric quantifications of cyclins D1, E, and B1 (**A**), and of apoptosis regulators p53, 27, p21, Bax (**B**), all of proteins normalized to β-actin. Data are the mean ± SD of three independent experiments. Student t test. * *p* < 0.05; ** *p* < 0.01; *** *p* < 0.001 versus Scr-siRNA.

The functional effects of PDE4D gene silencing on cell cycle and apoptosis were also confirmed by analysis of the expression of specific proteins. In particular, as shown in Figure 4A, the G0/G1 block of the cell cycle observed after PDE4D silencing was confirmed in HepG2 and Huh7 cells, which exhibited significantly increased levels of cyclin D1 and decreased levels of both cyclin E and B1, whereas in Hep3B cells the homeostasis disruption was confirmed by an inverse pattern characterized by the down-regulation of cyclin D1 and the up-regulation of cyclin E. Moreover, the expression of p27 and Bax was significantly increased in all HCC cells, while the interconnected pro-apoptotic p53/p21 were up-regulated only in HepG2 and Huh7 cells where these genes are expressed (Figure 4B).

### 3.3. PDE4D Silencing Modulates Cancer Related Genes in HCC Cells

The role of PDE4D in HCC was better determined by analysing the PDE4D-dependent expression with an Open Array Real-Time PCR platform of 624 cancer related genes in HepG2 and Huh7 cells silenced for PDE4D or control siRNA (PDE4D-siRNA; Scr-siRNA).

**Figure 5:**
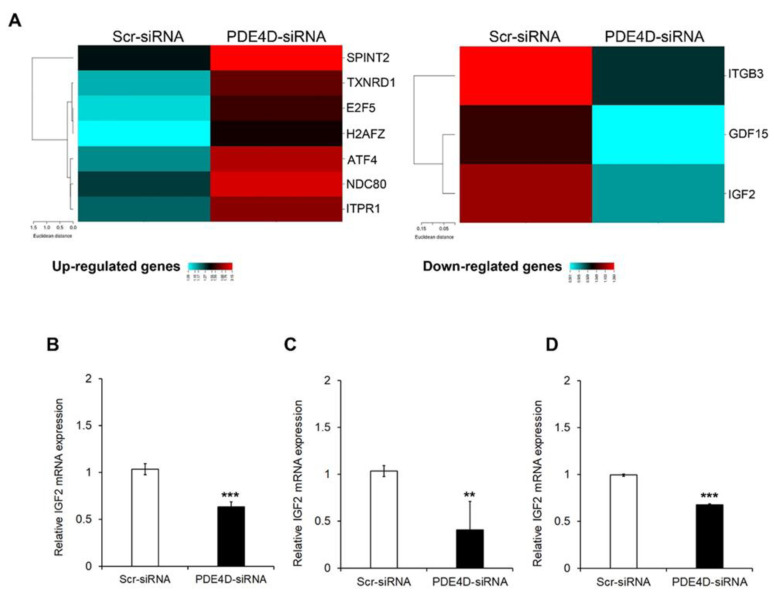
Gene expression analysis after transient silencing of PDE4D gene. (**A**) Heatmap representation of cancer-related genes analysed by TaqMan OpenArray that were up-regulated (left panel) or down-regulated (right panel) in HepG2 cells silenced or not silenced for PDE4D. This image was generated using online tools provided by CIMminer (http://discover.nci.nih.gov/cimminer/home.do, accessed on 23 February 2021). IGF2 gene expression analysis by qRT-PCR in HepG2 (**B**), Hep3B (**C**), and Huh7 (**D**) cells 48 h after transient silencing. Data are the mean ± SD of three independent experiments. Student t test. ** *p* < 0.01; *** *p* < 0.001 versus Scr-siRNA.

Gene expression profile analysis led to the identification of seven genes significantly up-regulated (fold change > 1.5) and 3 genes significantly down-regulated (fold change < 0.5) upon PDE4D depletion in HepG2 cells (Figure 5A). The same analysis in Huh7 cells revealed five significantly up-regulated genes (fold change > 1.5) and 284 significantly down-regulated genes (fold change < 0.5) after 48 h from transient silencing of PDE4D (Figure S5). The overlay of the up-regulated genes revealed that PDE4D silencing did not affect the expression of the same genes in the two cell lines, while the analysis of down-regulated genes highlighted that the mRNA expression levels of IGF2, a well-known epigenetic oncodriver in HCC [20], were significantly down-regulated in both HCC cell lines.

In order to validate these results, we performed a single qRT-PCR assay using an alternative probe for IGF2 in HepG2, Hep3B and Huh7 cells. As shown in Figure 5B–D, PDE4D silencing caused a significant down-regulation of IGF2 mRNA in all HCC cells. Moreover, as shown in Figure S6, the PDE4D silencing induced a down-regulation of the IGF2 protein amount.

### 3.4. Pharmacological Inhibition of PDE4D Represses Cell Growth, Induces Apoptosis and Down-Regulates IGF2 Transcription in HCC Cells

In order to evaluate if pharmacological inhibition of PDE4D may affect HCC oncogenic properties, the effect of different concentrations (1 μM, 5 μM, 10 μM, 50 μM, 100 μM) of a specific PDE4D inhibitor, Gebr-7b, was evaluated in HepG2 and Huh7 cells. As shown in Figure S7A, the dose of 5 μM exerted a reduction of cell viability of about 40% in HepG2 cells and 50% in Huh7 cells, at both 24 and 48 h. Gebr-7b, at the dose of 5 μM, was also able to increase intracellular cAMP levels in both HCC cell lines (Figure S7B). This concentration was thus used for all the subsequent experiments.

**Figure 6:**
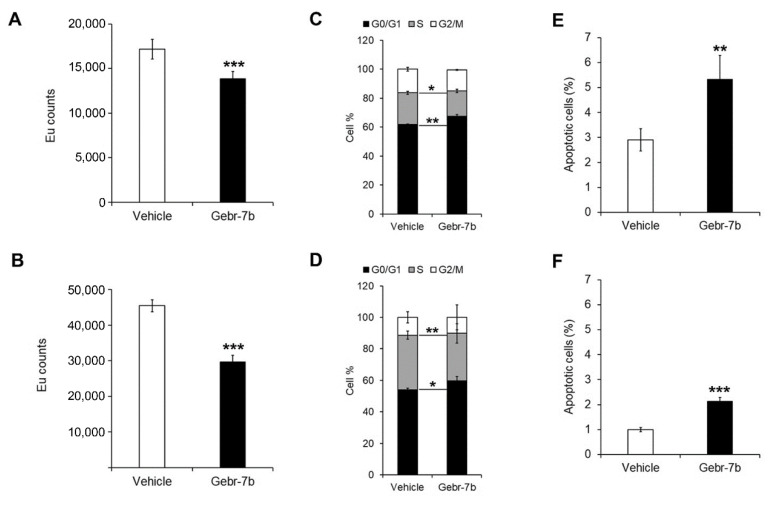
The effect of Gebr-7b on cell proliferation, cells cycle and apoptosis in HCC cells. Cell proliferation assay performed by DELFIA assay in HepG2 (**A**) and Huh7 (**B**) cells. Value expressed in EU counts. Cell cycle profiles measured by propidium iodide staining and flow cytometry analysis in HepG2 (**C**) and Huh7 (**D**) cells. Flow cytometry evaluation of apoptosis using Annexin V-FITC/PI staining in HepG2 (**E**) and Huh7 (**F**) cells. Data are the mean ± SD of three independent experiments. Student t test. * *p* < 0.05; ** *p* < 0.01; *** *p* < 0.001 versus vehicle.

Analysis of cell proliferation, assessed by BrdU, confirmed that the treatment with 5 μM Gebr-7b significantly reduced cell proliferation after 48 h of treatment in both cell lines compared to control cells (Figure 6A,B).

As shown in Figure 6C,D and Table S4, the cell cycle analysis by flow cytometry revealed that after 48 h with 5 μM Gebr-7b, HepG2 and Huh7 cells had a significantly decreased percentage of cells in S phase and increased percentage of cells in G0/G1 phase compared to vehicle-treated cells. As observed in HCC cells silenced for PDE4D, the pharmacological inhibition of PDE4D activity by 5 μM Gebr-7b caused a significant up-regulation of the apoptotic rate in both HepG2 and Huh7 cells (Figure 6E,F).

**Figure 7:**
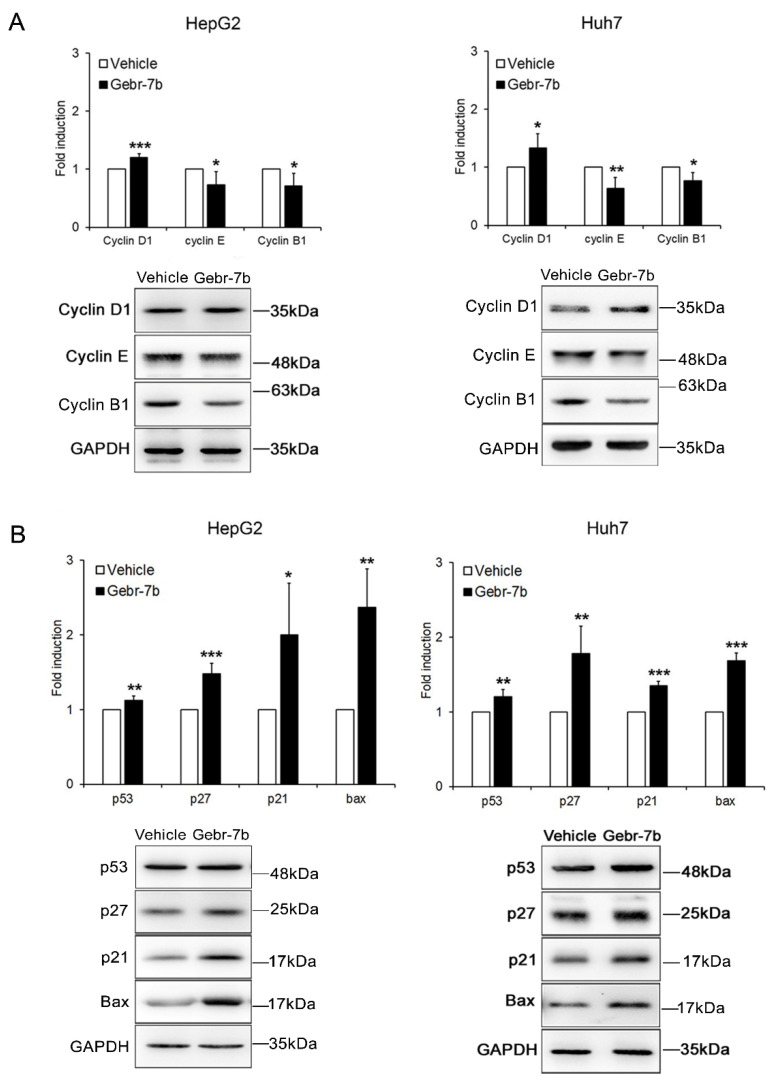
The effect of Gebr-7b on cell cycle regulators and apoptotic proteins. Western blot analysis of cell cycle (**A**) and apoptotic (**B**) proteins in HepG2, and Huh7 cells, 48 h after treatment with 5 μM Gebr-7b. The graphs represent densitometric quantifications of the specified proteins normalized to GAPDH. Data are mean ± SD of three independent experiments. Student t test. * *p* < 0.05; ** *p* < 0.01; *** *p* < 0.001 versus vehicle.

The Gebr-dependent G0/G1 arrest was further confirmed by a significant increase of cyclin D1 expression levels and decrease of cyclin E and B1 expression levels in both HCC cell lines (Figure 7A). The analysis of pro-apoptotic proteins, including p53, p21, p27, and Bax, confirmed apoptosis induction after treatment with Gebr-7b (Figure 7B).

**Figure 8:**
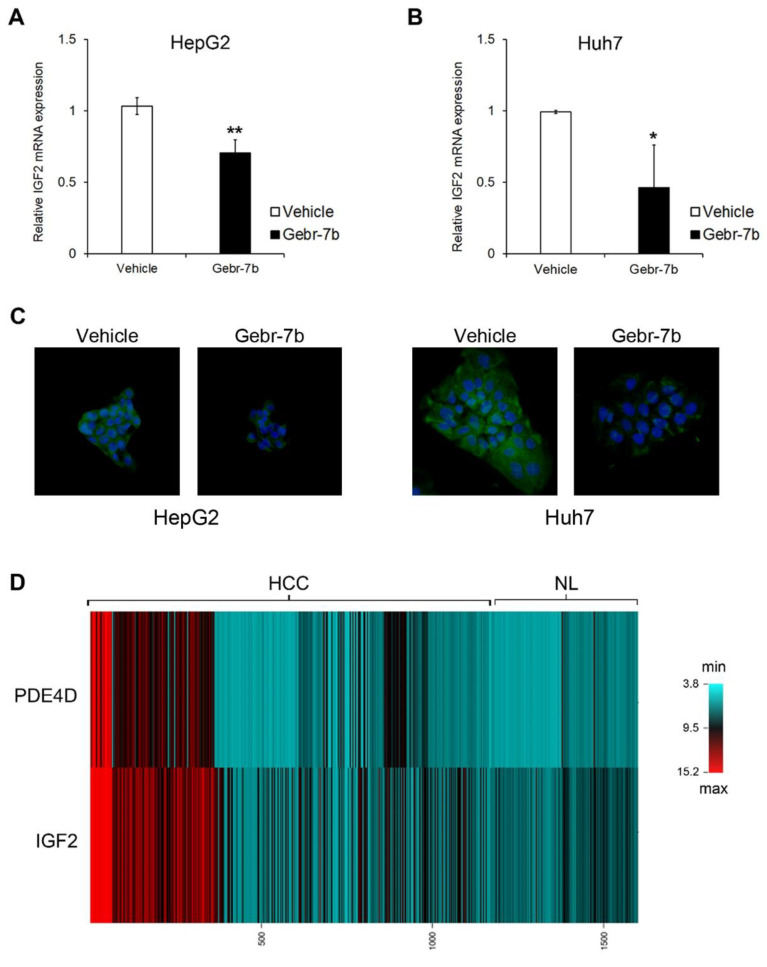
The effect of Gebr-7b on IGF2 gene/protein expression. IGF2 gene expression analysis by qRT-PCR in HepG2 (**A**), and Huh7 (**B**) cells after 48 h of treatment with 5 μM Gebr-7b. Data are the mean ± SD of three independent experiments. Student t test. * *p* < 0.05; ** *p* < 0.011 versus vehicle. (**C**) Representative immunofluorescence images of IGF2 protein (green). Nuclei were counterstained with DAPI (blue). Magnification 40×. (**D**) Heatmap representation of PDE4D and IGF2 expression of 1465 HCCs compared to 465 NL. This image was generated using online tools provided by CIMminer (http://discover.nci.nih.gov/cimminer/home.do, accessed on 23 February 2021).

Finally, as observed in HCC cells silenced for PDE4D, pharmacological inhibition of the protein activity caused a significant reduction of IGF2 gene transcription (Figure 8A,B) and protein levels (Figure 8C). This trend of correlation between PDE4D and IGF2 expression patterns was further confirmed by the analysis of gene expression data of 1165 HCCs and 465 NL retrieved from the CancerLivER database (Figure 8D) [21].

## 4. Discussion

In the present study we investigated, for the first time, the expression and modulation of PDE4D in HCC and we evaluated the potential antitumor effects of its depletion or inhibition in different HCC cells.

PDE4D is one of the PDE4 isoforms that was previously suggested as a potential oncogene in several human solid cancers, including lung, skin, breast, ovary, and prostate tumours [11,22,23,24,25], while there is no evidence of its role in HCC. In a previous study, Peng et al. [16] demonstrated that PDE4A expression was increased in HCC tissues compared to adjacent normal liver tissues and this pattern of over-expression was an indication of a bad prognosis for patients. Over-expression of PDE4A protein was also found in HepG2 cells compared to more differentiated cells [15]. Here, we demonstrated that also PDE4D protein was up-regulated in human HCC tissues and its expression progressively increased with the severity of the tumour. Moreover, the in silico gene expression analysis revealed a significant over-expression of the PDE4D transcript in HCC samples compared to non-tumour samples and, of note, higher levels of PDE4D gene expression were an index of poor prognosis. These results suggest a relationship between PDE4D and the degree of tumour aggressiveness, but, as they were obtained in small subsets of HCCs further investigations are needed.

Previous studies by Massimi et al. [15] showed that proliferating HepG2 cells have higher levels of cAMP-PDE activity and PDE4D protein expression than non-proliferating differentiated HepaRG cells. Here, we showed that PDE4D expression and activity were significantly up-regulated mainly in more aggressive HCC cells. PDE4D presents 9 splice variants (PDE4D1-9). From our data, the highest molecular weight variant (about 90 kDa) is the most up-regulated in tumorigenic cells. Data in the literature suggest that this splicing variant corresponds to PDE4D4 [26]. This isoform presents, at the N-terminal end, a PKA specific phosphorylation site implicated in the post-translational regulation of this protein, which is absent in the other isoforms. Phosphorylation, in fact, allows a conformational change of the enzyme with a consequent increase in its activity, affinity for magnesium ions and sensitivity to the PDE4 inhibitor rolipram [27].

Besides the potential role of PDE4D as a predictive factor in HCC prognosis, it could also represent a good candidate for targeted therapy in HCC. Indeed, we found that in vitro silencing of the PDE4D gene reduced its protein expression/activity, as well as moderately cell proliferation by promoting apoptosis and inducing the arrest of the cell cycle in G0/G1 phase, in HepG2 and Huh7, or in G2/M phase, in Hep3B cell lines. The anti-proliferative effects of PDE4D silencing had been previously reported in other types of cancer including lung cancer and prostate cancer [28,29]. Even though our data demonstrated that the gene silencing of PDE4D has produced favourable effects in vitro, the translation of such findings to the more complex clinical arena, as well as other clinical applications of RNA interference-dependent tools, remains an open issue due to siRNAs cellular toxicity and their complex delivery to specific tissues [30].

Hence, the use of drugs that act against PDE4D protein expression and/or activity is still strongly encouraged. The use of PDE4 inhibitors in various diseases has been extensively studied for the treatment of several clinical conditions, including pulmonary diseases [31,32], nervous system-related disorders [33,34] and different kinds of cancer [10,35,36,37]. Among the currently available PDE4D inhibitors, Gebr-7b is the most specific one [38]. This drug, highly potent and cell permeable, is of particular interest because it can be administered as an adjuvant to conventional therapies to reduce drug resistance in tumour models, such for breast cancer [39].

In the present study, the pharmacological inhibition of PDE4D by Gebr-7b resulted in an increase in cAMP levels and reduced HCC cell growth by anti-proliferative and pro-apoptotic effects. All the anti-tumorigenic in vitro effects of PDE4D depletion/inhibition are associated with the modulation of some key regulators of cell cycle and apoptosis, including cyclins D1, E and B1, p21, p27, Bax and p53. Although the relationship between these proteins and PDE4D inhibitors has been previously reported for other tumours [40], our findings represent the first evidence of their specific link with the PDE4D isoform in HCC.

Interestingly, our data highlighted that PDE4D silencing may affect the gene expression of several cancer-related genes in all HCC cell lines, among which emerged IGF2. This gene plays a crucial role in hepatocarcinogenesis and previous studies demonstrated that it is highly over-expressed in in vivo and in vitro HCC models [41,42,43]. Indeed, we found that IGF2 was down-regulated by both silencing and pharmacological inhibition of PDE4D, thus confirming the anti-tumorigenic effects of PDE4D protein depletion. Moreover, gene expression data, available in the CancerLivER data base (https://academic.oup.com/database/article/doi/10.1093/database/baaa012/5798989), accessed on 23 February 2021 confirmed that PDE4D over-expression in human HCCs correlated with an increased expression of IGF2.

This last finding suggests a possible molecular connection between PDE4D and IGF2. IGF2 transcription is subject to genomic imprinting, however there is extensive evidence for the dysregulation of this gene in human tumours including HCC, where it is epigenetically regulated [44]. Although the loss of imprinting regulation in cancer is believed to occur as a consequence of the hypermethylation of IGF2/H19lnc cluster, in HCC the cluster is hypomethylated. Indeed, IGF2 over-expression in HCC cells is often correlated to an unexpected up-regulation of H19lnc RNA and to an aberrant epigenetic regulation of the whole cluster [44]. Therefore, we hypothesize that PDE4D silencing may affect the transcription of the IGF2/H19 cluster by altering its epigenetic regulation with mechanisms that could involve different already reported H19lnc regulators including cAMP, PKA and paxillin [45,46]. However, since the up-stream signals for regulation of IGF2/H19 cluster are only partly known during hepatocarcinogenesis, our hypothesis deserves further exploration.

## 5. Conclusions

In conclusion, our study revealed that PDE4D is aberrantly over-expressed in HCCs, where it is a predictor of a more aggressive disease phenotype and poor prognosis, thus suggesting that this protein could be used as a biomarker for patient outcomes. Moreover, the depletion or inhibition of PDE4D blocked the growth of HCC cells, highlighting the role of PDE4D as an actionable drug targets for HCC therapy.

## Data Availability

The data presented in this study are available as Supplementary Materials and on request from the corresponding authors.

## Supplementary Materials

The following are available online at https://www.mdpi.com/article/10.3390/cancers13092182/s1, Table S1: List of used antibodies, Table S2: List of TaqMan OpenArray Human Cancer Panel probes, Table S3: Cell cycle distribution in Scr-siRNA and PDE4D-siRNA HCC cells, Table S4: Cell cycle distribution in Vehicle-treated and Gebr-7b-treated HCC cells, Figure S1: PDE4D expression levels in HCC samples compared to normal liver samples (NL), Figure S2: PDE4D protein expression in HCC cell lines, Figure S3: The effect of PDE4D silencing on the expression of PDE4D, PDE4A, PDE4B, and PDE4C mRNA in HCC cells, Figure S4: The effect of PDE4D silencing on its protein expression and activity in HCC cells, Figure S5: Gene expression analysis in Huh7 cells after silencing of PDED4 gene, Figure S6: Expression of IGF2 protein upon PDE4D silencing in HCC cells, Figure S7: Evaluation of the effects of Gebr-7b on HCC cell growth and PDE4D activity, Figure S8: Uncropped Western blot figures.
