# Supplementary material for: Phosphodiesterase 4D Depletion/Inhibition Exerts Anti-Oncogenic Properties in Hepatocellular Carcinoma
Source: Cancers (Basel). 2021 May 1;13(9):2182. doi: 10.3390/cancers13092182 (PMC8125776; doi:10.3390/cancers13092182)
Supplement: Supplementary file 1 [file cancers-13-02182-s001.zip › cancers-1183788-supplementary/cancers-1183788-Figure S8.docx]

**Supplementary Materials S8**

Used for figure Supplementary 2


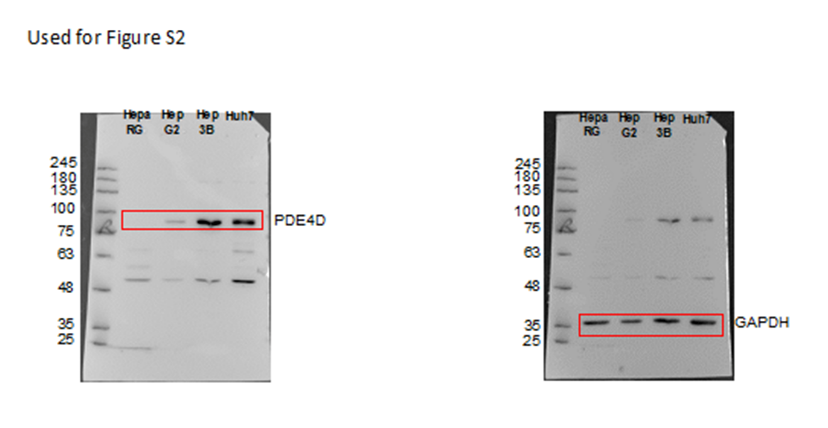


Used for figure Supplementary 3

**HepG2 and Hep3B**







**Scr**

**-siRNA**

**PDE4D**

**-siRNA**

**PDE4D**

**-siRNA**

**Scr**

**-siRNA**

**PDE4D**

**-siRNA**

**PDE4D**

**-siRNA**

**Scr**

**-siRNA**

**PDE4D**

**-siRNA**

**PDE4D**

**-siRNA**

**Scr**

**-siRNA**

**PDE4D**

**-siRNA**

**PDE4D**

**-siRNA**

**GAPDH**

**Huh7**

**PDE4D**

**-siRNA**

**Scr**

**-siRNA**





**PDE4D**

**GAPDH**

Used for figure 4

**HepG2**


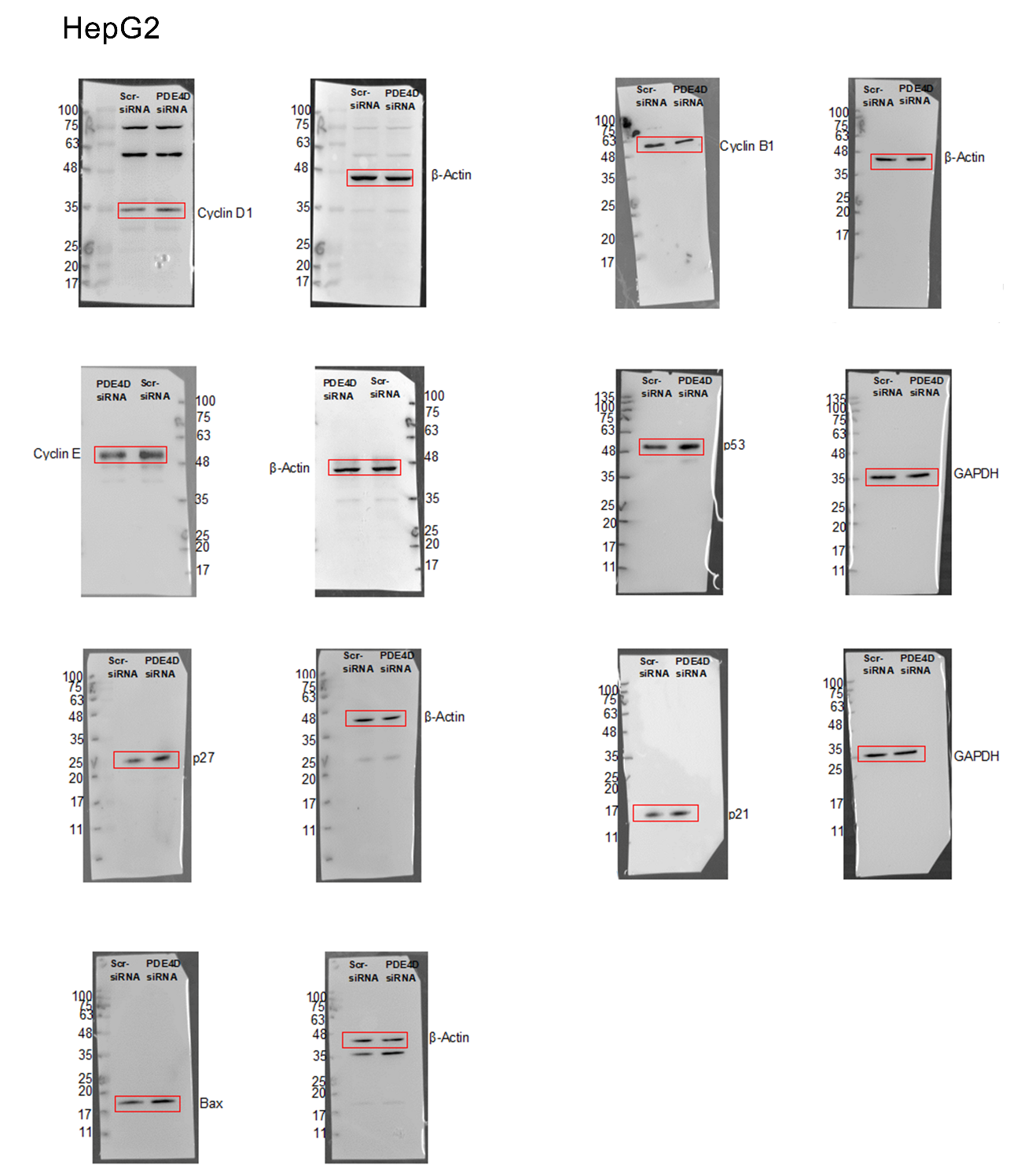


Used for figure 4

**Huh7**


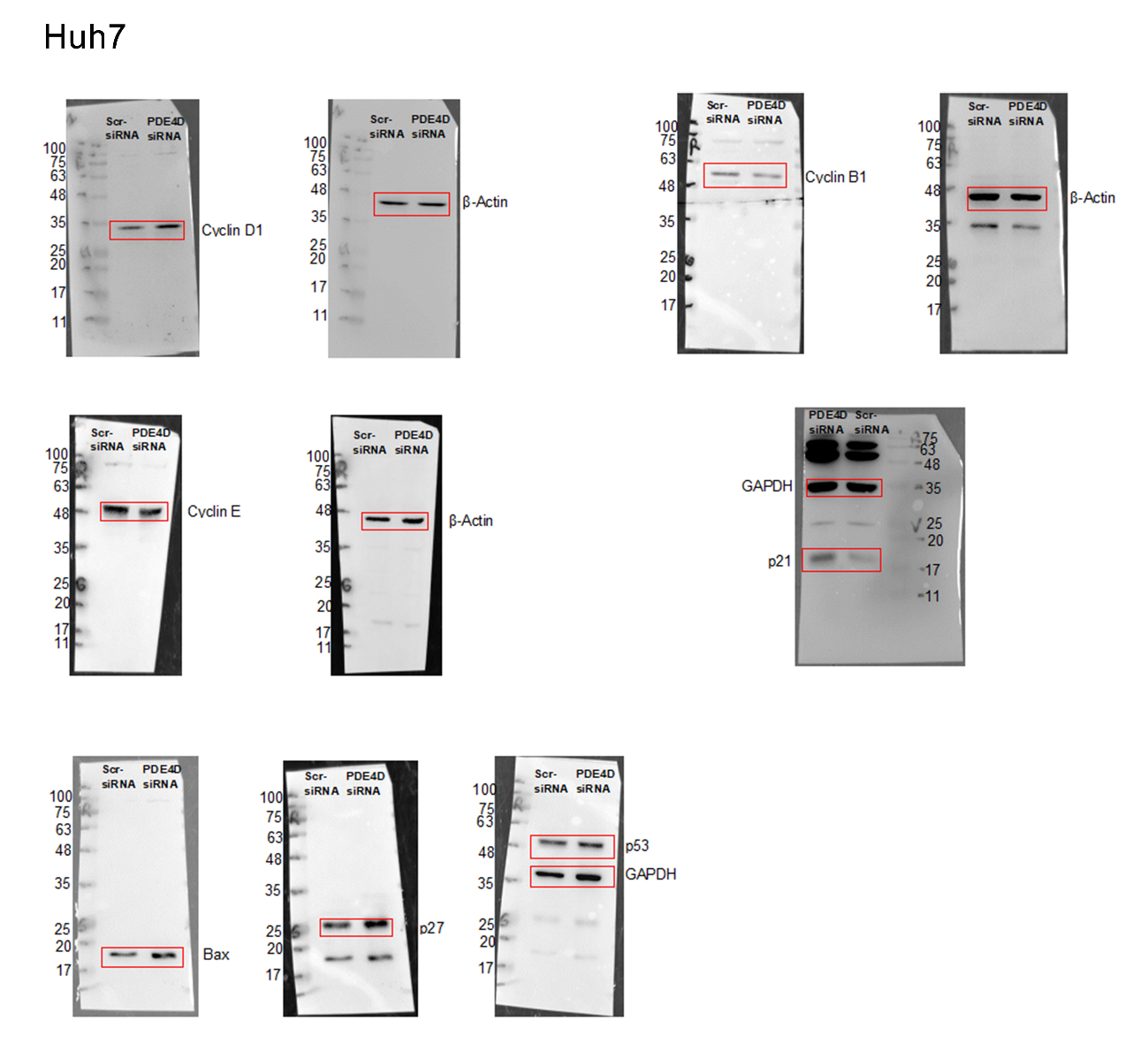


**Hep3B**


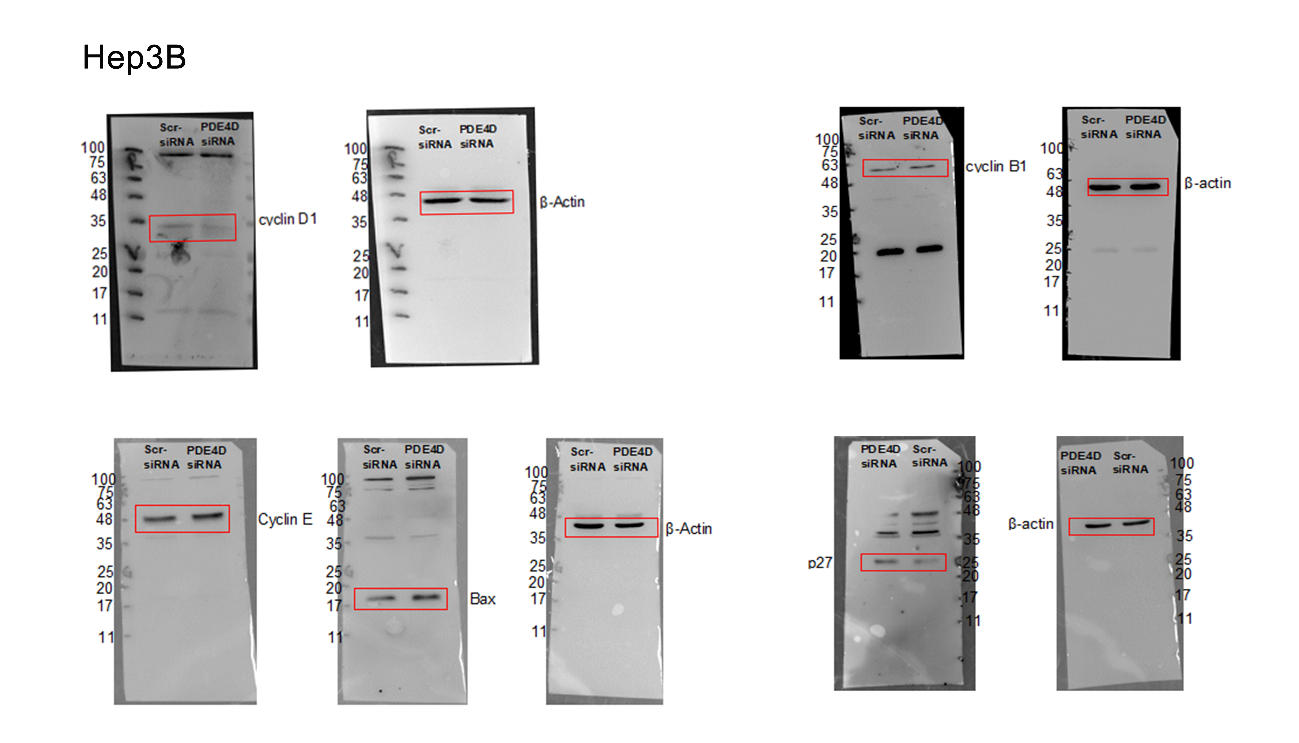


Used for figure 7

**HepG2**


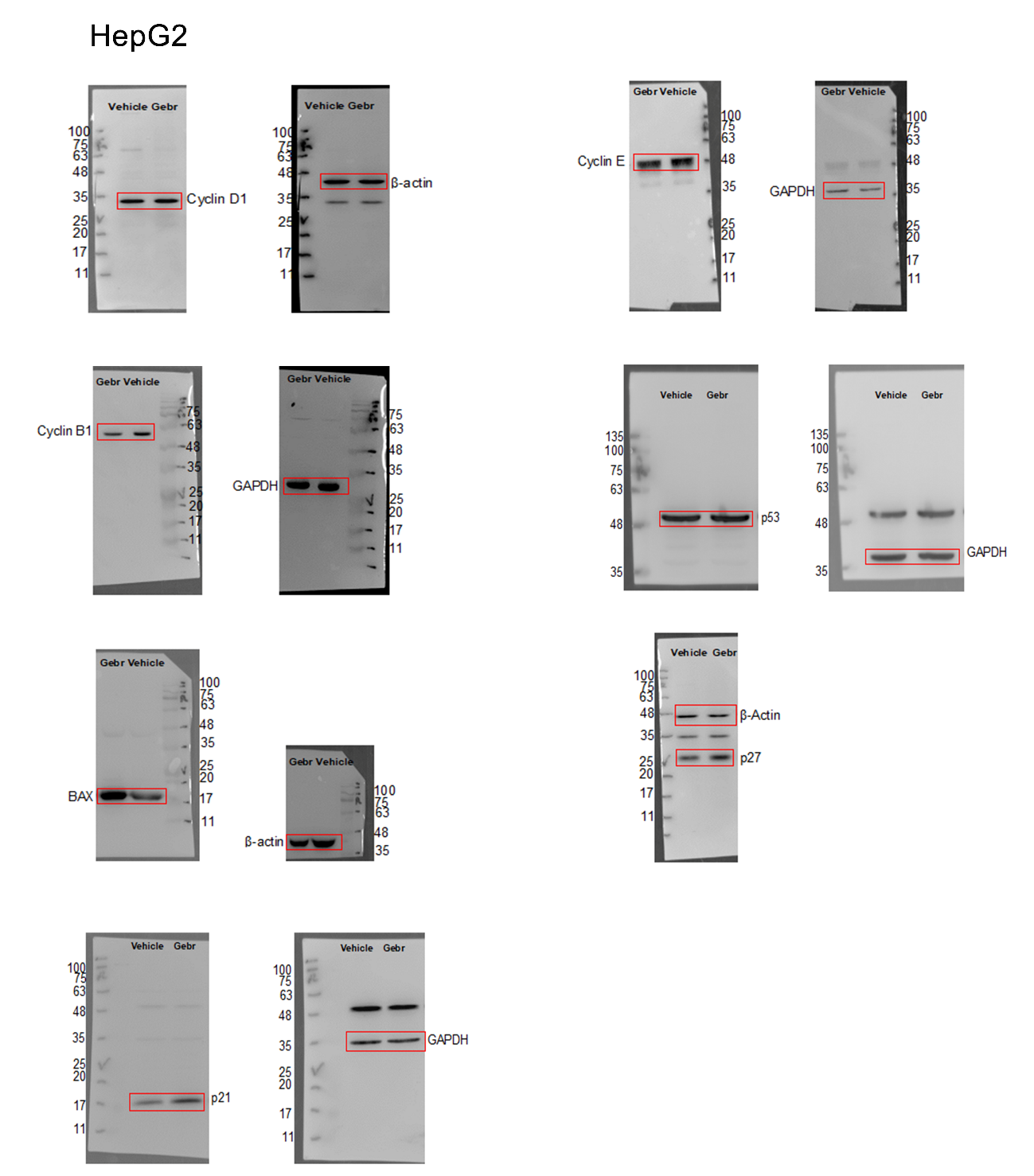


Used for figure 7

**Huh7**


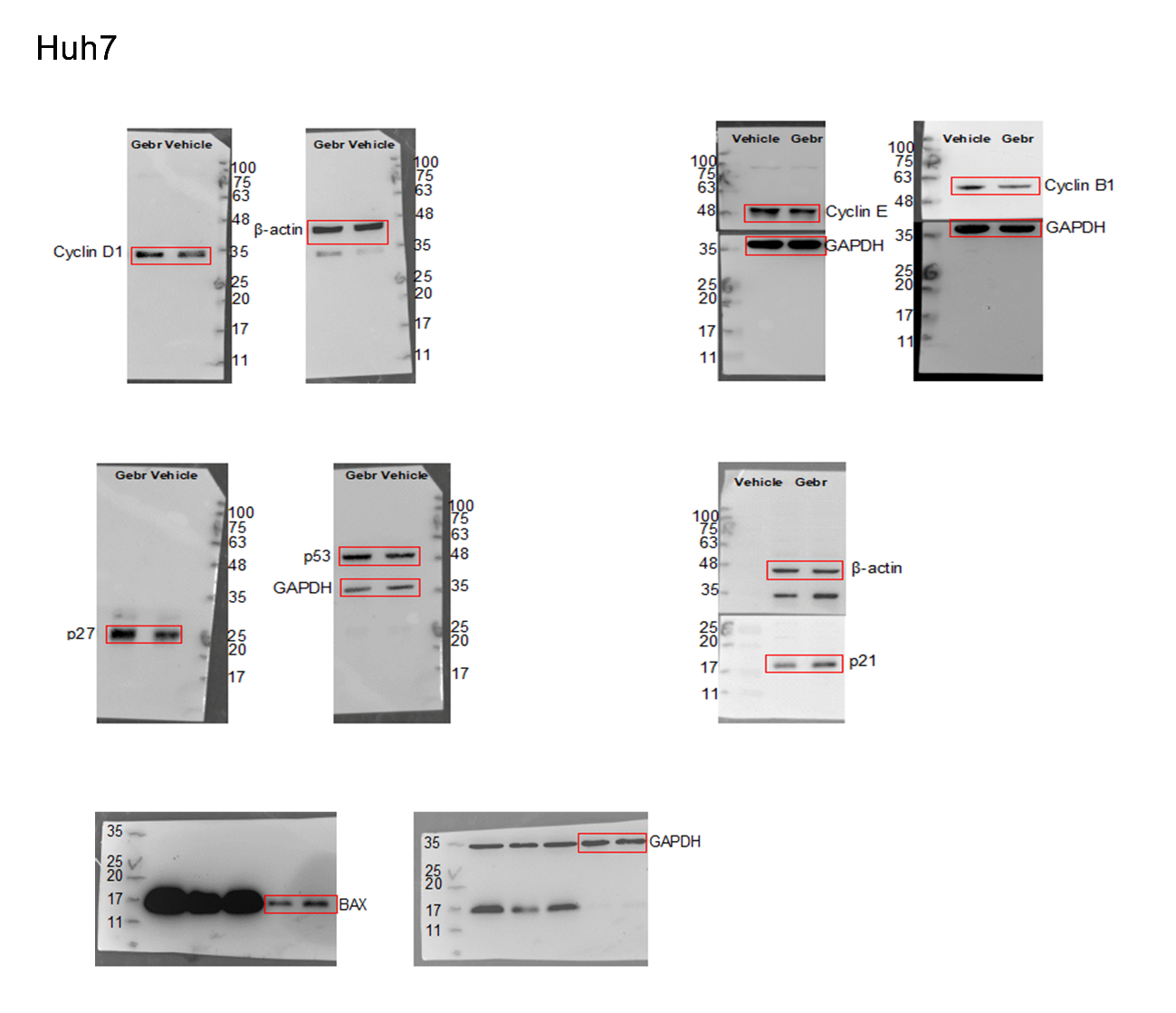


**Gebr Vehicle**

**Gebr Vehicle**
