# Supplementary material for: Phosphodiesterase 4D Depletion/Inhibition Exerts Anti-Oncogenic Properties in Hepatocellular Carcinoma
Source: Cancers (Basel). 2021 May 1;13(9):2182. doi: 10.3390/cancers13092182 (PMC8125776; doi:10.3390/cancers13092182)
Supplement: Supplementary file 1 [file cancers-13-02182-s001.zip › cancers-1183788-supplementary/cancers-1183788-supplementary.pdf]

Supplementary Materials

Table S1. List of used antibodies for WB.

	Antibody Name	Company	Dilution
WB	Mouse anti-GAPDH	Immunological Sciences (MAB-10578)	1:5000
	Rabbit anti-cyclin D1	Immunological Sciences (AB82346)	1:1000
	Mouse anti-cyclin E	Thermo Fisher Scientific (MA5-14336)	1.1000
	Mouse anti-Cyclin B1	Santa Cruz Biotechnology (sc-245)	1.1000
	Mouse anti-p53	Santa Cruz Biotechnology (sc-65344)	1:500
	Rabbit anti-Actin beta	Immunological Sciences (AB81599)	1.2000
	Rabbit anti-p21$^{waf1/cip1}$	Cell Signaling (2947)	1.1000
	Mouse anti-p27^{kip1}	Cell Signaling (SX53G8.5)	1:500
	Rabbit anti-Bax	Immunological Sciences (AB-82329)	1:1000
	Rabbit anti-PDE4D	FabGennix Inc. (PD4-400P)	1:1000
	Anti-rabbit peroxidase-conjungated	Jackson immunoresearch (111-035-003)	1.10000
	Anti-mouse peroxidase-conjungated	Jackson immunoresearch (111-035-003)	1.10000

Table S2. Probes included in TaqMan® OpenArray® Human Cancer Panel.

Assay ID	Gene	Target/Control
Hs00152928_m1	EGR1	Target
Hs00152933_m1	TLR3	Target
Hs00152939_m1	TLR4	Target
Hs00152971_m1	TLR7	Target
Hs00152973_m1	TLR9	Target
Hs00153074_m1	ROCK2	Target
Hs00153138_m1	CCNA2	Target
Hs00153277_m1	CDKN1B	Endogenous Control
Hs00153280_m1	TP53I3	Target
Hs00153294_m1	RELA	Target
Hs00153353_m1	BIRC5	Target
Hs00153380_m1	CCND2	Target
Hs00153418_m1	RAD51	Target
Hs00153451_m1	E2F1	Target
Hs00153510_m1	MME	Target
Hs00154054_m1	ANXA5	Target
Hs00154208_m1	CA9	Target

Hs00154457_m1	CIRBP	Target
Hs00155479_m1	CYR61	Target
Hs00156411_m1	CDC25C	Target
Hs00156454_m1	CEBPG	Target
Hs00156455_m1	CENPA	Target
Hs00157163_m1	CTPS	Target
Hs00157213_m1	CTSE	Target
Hs00157731_m1	GNAZ	Target
Hs00157817_m1	GRB2	Target
Hs00159081_m1	MATN3	Target
Hs00159092_m1	MDM4	Target
Hs00159210_m1	MNDA	Target
Hs00159600_m1	NID1	Target
Hs00159627_m1	NMBR	Target
Hs00160947_m1	PTPRN	Target
Hs00161707_m1	SKI	Target
Hs00161719_m1	SLC1A4	Target
Hs00161826_m1	SLC16A1	Target
Hs00162613_m1	TCF4	Target
Hs00163506_m1	ACADS	Target
Hs00163761_m1	BTK	Target
Hs00164004_m1	COL1A1	Target
Hs00164932_m1	ICAM1	Target
Hs00164940_m1	IDUA	Target
Hs00165949_m1	TIMP3	Target
Hs00166071_m1	BLMH	Target
Hs00166169_m1	G6PD	Endogenous Control
Hs00166223_m1	IFNGR1	Target
Hs00166294_m1	KRT2	Target
Hs00166467_m1	OXCT1	Target
Hs00167402_m1	ACY1	Target
Hs00168547_m1	NQO1	Target
Hs00169258_m1	F2R	Target
Hs00169407_m1	RIPK1	Target
Hs00169491_m1	STK3	Target
Hs00169714_m1	NF1	Target
Hs00169777_m1	PECAM1	Target
Hs00170630_m1	FOS	Target
Hs00170723_m1	TYRO3	Target
Hs00170780_m1	CIB1	Target
Hs00171022_m1	CXCL12	Target
Hs00171132_m1	GDF15	Target
Hs00171165_m1	OSM	Target
Hs00171172_m1	AR	Target
Hs00171191_m1	FBN1	Target
Hs00171254_m1	IGF2	Target
Hs00171375_m1	FES	Target
Hs00171558_m1	TIMP1	Target
Hs00171656_m1	EPHA2	Target
Hs00171832_m1	FGF8	Target
Hs00172187_m1	POLR2A	Endogenous Control
Hs00172870_m1	NR2F6	Target
Hs00172895_m1	ABI2	Target
Hs00173072_m1	TSG101	Target
Hs00173503_m1	FRZB	Target
Hs00173611_m1	KLK10	Target
Hs00173742_m1	FGF3	Target
Hs00173872_m1	ODZ1	Target
Hs00174029_m1	KIT	Target
Hs00174128_m1	TNF	Target
Hs00174141_m1	CD59	Target
Hs00174164_m1	CSF1	Target
Hs00174297_m1	CD70	Target
Hs00174474_m1	LAMP2	Target
Hs00174517_m1	NFKB2	Target
Hs00174674_m1	IER3	Target
Hs00174819_m1	SEMA4D	Target
Hs00174860_m1	ESR1	Target
Hs00174877_m1	LEP	Target
Hs00174970_m1	STC1	Target
Hs00175188_m1	CTSC	Target
Hs00175935_m1	CDK4	Target
Hs00175938_m1	CDKN1C	Target
Hs00176119_m1	AK1	Target
Hs00176222_m1	CDK9	Target
Hs00176258_m1	CSNK1G2	Target
Hs00176481_m1	CDKN2D	Target
Hs00176538_m1	ERBB3	Target
Hs00176628_m1	FYN	Target
Hs00176719_m1	LYN	Target
Hs00176815_m1	PAK1	Target
Hs00176839_m1	CDK17	Target
Hs00176884_m1	PDPK1	Target
Hs00176966_m1	PRKAR2B	Target

Hs00176973_m1	PRKCA	Target
Hs00176998_m1	PRKCB	Target
Hs00177010_m1	PRKCG	Target
Hs00177037_m1	PRKD1	Target
Hs00177051_m1	PRKCZ	Target
Hs00177357_m1	RPS6KB1	Target
Hs00177464_m1	TYK2	Target
Hs00177481_m1	AKAP1	Target
Hs00177522_m1	CDC42BPA	Target
Hs00177708_m1	TNK1	Target
Hs00177766_m1	CDKL1	Target
Hs00177774_m1	PKMYT1	Target
Hs00177821_m1	BUB1	Target
Hs00177957_m1	MAPKAPK3	Target
Hs00178289_m1	AKT1	Target
Hs00178297_m1	MAP3K8	Target
Hs00178340_m1	FGR	Target
Hs00178427_m1	LCK	Target
Hs00178455_m1	PRKCE	Target
Hs00178463_m1	ROCK1	Target
Hs00178494_m1	SRC	Target
Hs00178500_m1	TIE1	Target
Hs00178726_m1	MAP3K5	Target
Hs00178837_m1	CDK16	Target
Hs00178914_m1	PRKCD	Target
Hs00179382_m1	APPL1	Target
Hs00179683_m1	STK32B	Target
Hs00179843_m1	SHH	Target
Hs00179845_m1	MET	Target
Hs00180035_m1	NRAS	Target
Hs00180127_m1	DEK	Target
Hs00180269_m1	BAX	Target
Hs00180288_m1	CBLB	Target
Hs00180312_m1	MLLT3	Target
Hs00180437_m1	DCC	Target
Hs00180450_m1	GRB7	Target
Hs00180529_m1	WNT1	Target
Hs00180533_m1	ARHGEF5	Target
Hs00180562_m1	RBL2	Target
Hs00180679_m1	PIK3CA	Target
Hs00181051_m1	APC	Target
Hs00181211_m1	IGFBP3	Target
Hs00181419_m1	IGF2R	Target
Hs00181605_m1	ADM	Target
Hs00181767_m1	IGFBP4	Target
Hs00181881_m1	ITPR1	Target
Hs00182059_m1	MCC	Target
Hs00182082_m1	MYD88	Target
Hs00182192_m1	PLCG2	Target
Hs00182314_m1	RASGRF1	Target
Hs00182370_m1	SHB	Target
Hs00182558_m1	TRADD	Target
Hs00182896_m1	DVL1	Target
Hs00183533_m1	IPO8	Endogenous Control
Hs00183573_m1	GNA13	Target
Hs00183624_m1	NMU	Target
Hs00183953_m1	BCAR1	Target
Hs00184427_m1	BARD1	Target
Hs00184500_m1	ABCB1	Target
Hs00185390_m1	BAG1	Target
Hs00185574_m1	EZR	Target
Hs00185584_m1	VIM	Target
Hs00186374_m1	PIR	Target
Hs00186419_m1	BHLHE40	Target
Hs00186447_m1	DEGS1	Target
Hs00186495_m1	TMEFF1	Target
Hs00186689_m1	ALDH4A1	Target
Hs00187740_m1	PRC1	Target
Hs00188713_m1	BAG3	Target
Hs00189535_m1	FUT8	Target
Hs00191535_m1	CLTC	Target
Hs00191583_m1	LITAF	Target
Hs00191882_m1	DHX8	Target
Hs00192202_m1	MARS	Target
Hs00192399_m1	PIK3CD	Target
Hs00192574_m1	SFPQ	Target
Hs00192581_m1	SIAH2	Target
Hs00193725_m1	XRCC3	Target
Hs00193920_m1	CHAF1A	Target
Hs00194421_m1	ALDH6A1	Target
Hs00195432_m1	SMAD1	Target
Hs00195504_m1	MCM6	Target
Hs00195612_m1	TBX3	Target
Hs00196101_m1	NDC80	Target

Hs00196132_m1	PRAME	Target
Hs00196146_m1	PECI	Target
Hs00196542_m1	PPP2R5A	Target
Hs00196708_m1	SFRS7	Target
Hs00197328_m1	HYOU1	Target
Hs00197394_m1	PRDX4	Target
Hs00197646_m1	TBL3	Target
Hs00197856_m1	SARS	Target
Hs00197982_m1	BCL2L11	Target
Hs00198091_m1	CGRRF1	Target
Hs00198320_m1	PLK2	Target
Hs00198887_m1	BTG2	Target
Hs00199831_m1	ESM1	Target
Hs00200720_m1	PHB2	Target
Hs00201046_m1	HYAL1	Target
Hs00201226_m1	CASC3	Endogenous Control
Hs00202756_m1	SEC14L2	Target
Hs00204173_m1	MRPL13	Target
Hs00204205_m1	ATAD2	Target
Hs00204876_m1	ORC6L	Target
Hs00205182_m1	SND1	Target
Hs00205854_m1	RHOD	Target
Hs00208408_m1	KIF14	Target
Hs00211096_m1	CAMK2A	Target
Hs00212390_m1	LEF1	Target
Hs00212828_m1	PRKD2	Target
Hs00216455_m1	ECT2	Target
Hs00217947_m1	FGD6	Target
Hs00220127_m1	MCCC1	Target
Hs00220348_m1	CHPT1	Target
Hs00220393_m1	MGST1	Target
Hs00221277_m1	SCUBE2	Target
Hs00221499_m1	KAT2A	Target
Hs00221664_m1	DLG3	Target
Hs00221707_m1	XRCC5	Target
Hs00225132_m1	RASL11B	Target
Hs00229135_m1	WNT3	Target
Hs00230762_m1	GPR39	Target
Hs00231092_m1	E2F5	Target
Hs00231101_m1	ETV6	Target
Hs00231279_m1	REL	Target
Hs00231476_m1	TFAP2C	Target
Hs00231535_m1	CNBP	Target
Hs00231780_m1	EGR3	Target
Hs00231838_m1	MLLT10	Target
Hs00231908_m1	KDM5A	Target
Hs00232013_m1	FOSL2	Target
Hs00232074_m1	MYCN	Target
Hs00232399_m1	RELB	Target
Hs00232479_m1	LZTR1	Target
Hs00233492_m1	CANX	Target
Hs00233722_m1	ITGA3	Target
Hs00233790_m1	ITGAV	Target
Hs00234046_m1	PLCG1	Target
Hs00234119_m1	RAF1	Target
Hs00234140_m1	CCL2	Target
Hs00234244_m1	TGFB2	Target
Hs00234253_m1	TGFBR2	Target
Hs00234508_m1	MTOR	Target
Hs00234579_m1	MMP9	Target
Hs00234753_m1	MDM2	Target
Hs00234864_m1	HMMR	Target
Hs00236216_m1	ITGB4	Target
Hs00236329_m1	BCL2L1	Target
Hs00236810_m1	CP	Target
Hs00236884_m1	CSF3	Target
Hs00236949_m1	CCND3	Target
Hs00237047_m1	YWHAZ	Endogenous Control
Hs00237119_m1	MMP14	Target
Hs00241111_m1	FGFR1	Target
Hs00241497_m1	KITLG	Target
Hs00241844_m1	SERPINH1	Target
Hs00242386_m1	CBFB	Target
Hs00242728_m1	LIMK1	Target
Hs00242993_m1	PFKP	Target
Hs00243140_m1	ARID4A	Target
Hs00243327_m1	XRCC4	Target
Hs00243533_m1	CDK13	Target
Hs00247620_m1	NRG1	Target
Hs00248075_m1	BBC3	Target
Hs00248408_m1	40792	Target
Hs00248563_m1	PLCB1	Target
Hs00253876_m1	NR1D1	Target
Hs00254392_m1	EGLN1	Target

Hs00255374_m1	UCHL5	Target
Hs00255603_m1	GNAS	Target
Hs00259932_m1	SELENBP1	Target
Hs00259967_m1	PPP1R1B	Target
Hs00262861_m1	CDK4	Target
Hs00263393_m1	GTF2I	Target
Hs00264721_m1	MSH6	Target
Hs00264877_m1	PLG	Target
Hs00266592_m1	FBN2	Target
Hs00267157_s1	MAS1	Target
Hs00267597_m1	PRKAR1A	Target
Hs00268060_m1	MAPK12	Target
Hs00268847_m1	USP7	Target
Hs00268954_s1	FZD9	Target
Hs00269428_m1	PEA15	Target
Hs00269492_m1	TNFRSF10A	Target
Hs00269500_m1	GMPS	Target
Hs00269660_s1	RHOB	Target
Hs00269944_m1	BRAF	Target
Hs00269977_m1	COX6C	Target
Hs00270282_m1	STX1A	Target
Hs00270424_m1	CCNB2	Target
Hs00270514_m1	LONP1	Target
Hs00270768_m1	TLE1	Target
Hs00272002_m1	GNB2L1	Target
Hs00272649_s1	TPBG	Target
Hs00273319_m1	FKBP8	Target
Hs00273500_m1	STAT5B	Target
Hs00273561_s1	CD24;CD24L4	Target
Hs00275226_m1	EVL	Target
Hs00275656_m1	GSK3B	Target
Hs00276002_m1	SIVA1	Target
Hs00277090_m1	PIK3CG	Target
Hs00277190_s1	JUN	Target
Hs00286908_m1	KIF21A	Target
Hs00292627_s1	ZMYND8	Target
Hs00332674_m1	UHMK1	Target
Hs00355045_m1	CTNNB1	Target
Hs00355049_m1	CTNNB1	Target
Hs00355782_m1	CDKN1A	Endogenous Control
Hs00356079_m1	GSTM3	Target
Hs00357608_m1	RHOA	Target
Hs00357717_m1	CYC1	Target
Hs00357891_s1	JUNB	Target
Hs00358991_g1	CDK5	Target
Hs00359394_g1	MT3	Target
Hs00359840_m1	SLC2A3	Target
Hs00360961_m1	MAP2K2	Target
Hs00361432_s1	FZD2	Target
Hs00361747_m1	SORT1	Target
Hs00361869_g1	FZD5	Target
Hs00362370_m1	RGS19	Target
Hs00364282_m1	KRAS	Target
Hs00364293_m1	CDK1	Target
Hs00365098_m1	COL6A3	Target
Hs00365249_m1	CDKN2B	Target
Hs00365573_m1	WISP1	Target
Hs00365799_m1	CAMK2B	Target
Hs00365950_g1	HLA-G	Target
Hs00366152_m1	UBE2D2	Endogenous Control
Hs00366278_m1	TNFRSF10B	Target
Hs00366363_m1	CRHR1	Target
Hs00366497_m1	ETV3	Target
Hs00366502_g1	GNB2	Target
Hs00366726_m1	RAD21	Target
Hs00366740_m1	PPIH	Target
Hs00370265_m1	ASNS	Target
Hs00374226_m1	NR4A1	Target
Hs00374280_m1	STAT3	Target
Hs00377726_m1	PTGS1	Target
Hs00379185_m1	MIB1	Target
Hs00381676_m1	MLF1IP	Target
Hs00382884_m1	HSPA4	Target
Hs00382970_m1	PFDN5	Target
Hs00383065_m1	IP6K2	Target
Hs00384387_m1	CDK18	Target
Hs00385075_m1	MAPK3	Target
Hs00387426_m1	MAP2K4	Target
Hs00411505_m1	ASPM	Target
Hs00412445_m1	GCN1L1	Target
Hs00412720_m1	SP1	Target
Hs00413032_m1	TCF3	Target
Hs00413861_m1	KRT9	Target
Hs00415851_g1	CDC20	Target

Hs00416477_m1	TNK2	Target
Hs00426191_m1	HADHA	Target
Hs00426694_m1	AP2B1	Target
Hs00426889_m1	ATP5O	Target
Hs00427214_g1	PCNA	Target
Hs00427274_m1	PRKACA	Target
Hs00427469_m1	RFC4	Target
Hs00427621_m1	TBP	Target
Hs00427993_m1	AGFG1	Target
Hs00428286_g1	ELK1	Target
Hs00428633_m1	TUBA4A	Target
Hs00531110_m1	FAS	Target
Hs00533490_m1	SOD1	Target
Hs00533560_m1	TNFRSF1A	Target
Hs00539278_m1	MYRIP	Target
Hs00559413_m1	ANXA7	Target
Hs00559623_m1	MAPK13	Target
Hs00559804_m1	CAPN1	Target
Hs00560089_m1	TRAM1	Target
Hs00603745_mH	CCT5	Target
Hs00603799_m1	ARMC1	Target
Hs00605457_m1	E2F3	Target
Hs00605615_mH	MAP2K1	Target
Hs00607475_m1	GPR126	Target
Hs00608023_m1	BCL2	Target
Hs00608187_m1	TGFA	Target
Hs00608224_m1	WNT2	Target
Hs00608387_m1	NDRG1	Target
Hs00609073_m1	BRCA2	Target
Hs00609162_m1	EXT1	Target
Hs00609186_m1	GBE1	Target
Hs00609293_g1	HMBS	Target
Hs00609297_m1	HMBS	Endogenous Control
Hs00609566_m1	IGF1R	Target
Hs00609836_m1	AARS	Target
Hs00610263_m1	DVL3	Target
Hs00610314_m1	HDGF	Target
Hs00610318_m1	TGFBR1	Target
Hs00610483_m1	HRAS	Target
Hs00610488_m1	TP53BP2	Target
Hs00705137_s1	IFITM1	Target
Hs00705164_s1	SOCS1	Target
Hs00736972_m1	YES1	Target
Hs00737028_m1	DVL1	Target
Hs00737079_m1	GPR180	Target
Hs00740298_g1	HLA-C	Target
Hs00741586_mH	CDC42	Target
Hs00742533_s1	TUBB2A	Target
Hs00743792_s1	PABPC1	Target
Hs00746337_s1	PRKX	Target
Hs00747110_s1	RHOC	Target
Hs00747379_m1	ID2	Target
Hs00749309_s1	SYNCRIP	Target
Hs00754870_s1	DCN	Target
Hs00757122_m1	DDX10	Target
Hs00757841_m1	MTDH	Target
Hs00761767_s1	KRT19	Target
Hs00762869_s1	PKM2	Target
Hs00765553_m1	CCND1	Target
Hs00765730_m1	NFKB1	Target
Hs00793604_m1	YWHAB	Target
Hs00797747_s1	PFDN4	Target
Hs00799096_s1	RALBP1	Target
Hs00811070_m1	MAX	Target
Hs00818252_g1	KPNA2	Target
Hs00822401_m1	CTSL2	Target
Hs00824723_m1	UBC	Endogenous Control
Hs00829210_s1	MAP2K2;LOC407835	Target
Hs00830226_gH	FTL	Target
Hs00830594_s1	TFDP1	Target
Hs00854538_g1	PA2G4	Target
Hs00854841_g1	DEPDC1	Target
Hs00855044_g1	PHB	Target
Hs00855332_g1	LDHA	Target
Hs00869394_s1	ARHGAP5	Target
Hs00894816_g1	FASTK	Target
Hs00895608_m1	MX1	Target
Hs00898625_g1	YBX1	Target
Hs00899658_m1	MMP1	Target
Hs00900055_m1	VEGFA	Target
Hs00903413_m1	CYP19A1	Target
Hs00905030_m1	MYC	Target
Hs00907398_m1	MCM4	Target
Hs00907966_m1	PIK3CA	Target

Hs00909569_g1	ATF4	Target
Hs00910225_m1	ALB	Target
Hs00911250_m1	CSF1R	Target
Hs00912963_m1	CRAT	Target
Hs00914163_m1	GGH	Target
Hs00914334_m1	LTF	Target
Hs00917067_m1	TXNRD1	Target
Hs00918009_g1	GRB7	Target
Hs00920554_m1	MYB	Target
Hs00923894_m1	CDKN2A	Target
Hs00925195_m1	PRKCA	Target
Hs00927728_m1	PIK3CB	Target
Hs00931450_m1	SLC7A1	Target
Hs00932747_m1	TGFBI	Target
Hs00936301_m1	LRPAP1	Target
Hs00939627_m1	GUSB	Target
Hs00942543_m1	MYBL2	Target
Hs00943178_g1	PGK1	Target
Hs00943652_m1	ABL2	Target
Hs00944025_m1	CEACAM5	Target
Hs00944794_m1	CTNNA1	Target
Hs00945948_m1	RFC2	Target
Hs00946084_g1	HSPA5	Target
Hs00947433_m1	CTSB	Target
Hs00947994_m1	CDC25A	Target
Hs00950344_m1	SNAI2	Target
Hs00951083_m1	TFRC	Target
Hs00951941_m1	ETV1	Target
Hs00952642_m1	ANPEP	Target
Hs00953523_m1	MSH2	Target
Hs00955525_m1	ERBB4	Target
Hs00955802_m1	RPN2	Target
Hs00959834_m1	XRCC1	Target
Hs00960227_m1	MS4A7	Target
Hs00960942_m1	SPP1	Target
Hs00961554_m1	INSR	Target
Hs00961748_m1	TNFRSF1B	Target
Hs00962908_m1	THBS1	Target
Hs00963605_m1	TFDP2	Target
Hs00964426_m1	PDGFA	Target
Hs00964965_m1	TUBB3	Target
Hs00965587_m1	SLC20A1	Target
Hs00965889_m1	NOTCH4	Target
Hs00966302_m1	NF2	Target
Hs00966522_m1	PDGFB	Target
Hs00968295_m1	MMP11	Target
Hs00968308_m1	MMP3	Target
Hs00968436_m1	REL	Target
Hs00968567_s1	SFN	Target
Hs00969289_m1	BNIP3	Target
Hs00969569_m1	ATP5B	Endogenous Control
Hs00971475_m1	HSPH1	Target
Hs00977140_m1	RARB	Target
Hs00978565_m1	DTL	Target
Hs00981572_m1	RAB6B	Target
Hs00982607_m1	NINJ1	Target
Hs00983323_m1	FBP1	Target
Hs00984230_m1	B2M	Target
Hs00985157_m1	UCHL1	Target
Hs00988717_m1	ABCC4	Target
Hs00989970_m1	PRKCQ	Target
Hs00990023_m1	RAD50	Target
Hs00990732_m1	CD34	Target
Hs00991290_m1	RAB5A	Target
Hs00995282_g1	XRCC6	Target
Hs00997938_m1	GSK3A	Target
Hs00998018_m1	PDGFRA	Target
Hs00998133_m1	TGFB1	Target
Hs00998426_m1	CAPNS1	Target
Hs00998537_m1	WNT5A	Target
Hs01001469_m1	ITGB3	Target
Hs01001580_m1	ERBB2	Target
Hs01003267_m1	HPRT1	Endogenous Control
Hs01008571_m1	LCN2	Target
Hs01009038_m1	TCF7L2	Target
Hs01013123_m1	STAT2	Target
Hs01013989_m1	STAT1	Target
Hs01014511_m1	TLR2	Target
Hs01016789_m1	EZH2	Target
Hs01018151_m1	CASP8	Target
Hs01019082_mH	VDAC1	Target
Hs01020031_m1	MMP28	Target
Hs01020073_m1	TRIP13	Target
Hs01023087_m1	DHCR7	Target

Hs01023894_m1	CDH1	Target
Hs01025984_m1	RAC1	Target
Hs01026536_m1	CCNE1	Target
Hs01026983_m1	JAK1	Target
Hs01027360_g1	PSMA1	Target
Hs01027515_gH	STMN1	Target
Hs01028811_g1	EIF5	Target
Hs01030097_m1	CCNB1	Target
Hs01032443_m1	MKI67	Target
Hs01032845_m1	SEPP1	Target
Hs01032884_m1	RAC2	Target
Hs01033129_m1	STMN1	Target
Hs01034249_m1	TP53	Target
Hs01037584_m1	AP2M1	Target
Hs01040726_m1	DCK	Target
Hs01042010_m1	RELA	Target
Hs01042313_m1	TNFRSF1A	Target
Hs01045418_m1	SKIL	Target
Hs01046616_m1	TMEM45A	Target
Hs01047580_m1	STAT3	Target
Hs01048812_g1	CKS2	Target
Hs01050719_m1	NOTCH2	Target
Hs01052296_m1	IGFBP5	Target
Hs01052961_m1	FLT1	Target
Hs01053790_m1	ABCG2	Target
Hs01054576_m1	FOXO1	Target
Hs01055971_m1	LAMB1	Target
Hs01061576_m1	DHRS2	Target
Hs01062014_m1	NOTCH1	Target
Hs01062125_m1	TK1	Target
Hs01063168_m1	AXIN2	Target
Hs01064444_m1	AXL	Target
Hs01070442_m1	SPINT2	Target
Hs01072069_g1	RRM2	Target
Hs01072206_m1	RAB27B	Target
Hs01075861_m1	CD44	Target
Hs01076078_m1	EGFR	Target
Hs01078066_m1	RB1	Target
Hs01085579_m1	EIF2C2	Target
Hs01085691_m1	PTN	Target
Hs01086000_m1	TGFB3	Target
Hs01087307_m1	KLK13	Target
Hs01090047_m1	PRKCD	Target
Hs01090242_m1	SMO	Target
Hs01091564_m1	MCM2	Target
Hs01092205_g1	RAP1A	Target
Hs01093019_m1	GSPT1	Target
Hs01098873_m1	COL4A2	Target
Hs01099594_m1	BMP6	Target
Hs01099999_m1	EGF	Target
Hs01103749_m1	WT1	Target
Hs01104728_m1	ABL1	Target
Hs01106440_m1	MELK	Target
Hs01108847_m1	MMP17	Target
Hs01112307_m1	ATM	Target
Hs01114253_m1	TGFBR3	Target
Hs01115513_m1	PPARG	Target
Hs01117001_m1	CUL1	Endogenous Control
Hs01120030_m1	RET	Target
Hs01121102_g1	MAPRE1	Target
Hs01121709_m1	TSG101	Target
Hs01122781_m1	KIF3B	Target
Hs01124302_m1	M6PR	Target
Hs01125548_m1	UBE2L6	Target
Hs01547282_m1	IRF3	Target
Hs01548508_m1	MAPK8	Target
Hs01548727_m1	MMP2	Target
Hs01548894_m1	CDK2	Target
Hs01549976_m1	FN1	Target
Hs01550934_m1	CDC25B	Target
Hs01551876_m1	TJP1	Target
Hs01554450_m1	ERCC3	Target
Hs01554513_g1	MAD2L1	Target
Hs01555410_m1	IL1B	Target
Hs01556193_m1	BRCA1	Target
Hs01556702_m1	PGR	Target
Hs01568269_m1	APC	Target
Hs01568507_g1	RBBP4	Target
Hs01573874_g1	NME3	Target
Hs01582072_m1	AURKA	Target
Hs01588973_m1	CYCS	Target
Hs01591130_m1	TRRAP	Target
Hs01635324_s1	HMGN2	Target
Hs01652418_m1	COX7A2	Target

Hs01652468_g1	PGAM1	Target
Hs01683591_g1	HSPA8	Target
Hs01888362_g1	H2AFZ	Target
Hs01902432_s1	RAC1	Target
Hs01910190_s1	CARD17	Target
Hs01920617_s1	PRNP	Target
Hs01920652_s1	PTEN	Target
Hs01921749_s1	SPRY2	Target
Hs01941416_g1	KRT18	Target
Hs02330233_u1	JUND	Target
Hs02330328_s1	SOCS3	Target
Hs02339479_g1	NPM1	Target
Hs02339492_g1	PTMA	Target
Hs02621161_s1	NME1	Target
Hs02621185_s1	HDAC1	Target
Hs02621230_s1	PTEN	Target
Hs02621289_g1	TPT1	Target
Hs02741908_m1	INS	Target
Hs03044127_g1	HSPB1	Target
Hs03044281_g1	YWHAZ	Target
Hs03044902_g1	PRDX2	Target
Hs03063352_s1	BIRC5	Target
Hs03070834_m1	FBXO5	Target
Hs03676575_s1	ID1	Target
Hs03929097_g1	GAPDH	Target
Hs03986111_s1	TOB1	Target
Hs04194521_s1	PPIA	Target
Hs99999003_m1	MYC	Target
Hs99999008_m1	MDM2	Target
Hs99999018_m1	BCL2	Target
Hs99999141_s1	JUN	Target
Hs99999188_m1	CCNB1	Target
Hs99999901_s1	18S	Endogenous Control
Hs99999902_m1	RPLP0	Endogenous Control
Hs99999903_m1	ACTB	Endogenous Control
Hs99999904_m1	PPIA	Endogenous Control
Hs99999905_m1	GAPDH	Endogenous Control
Hs99999906_m1	PGK1	Endogenous Control
Hs99999907_m1	B2M	Endogenous Control
Hs99999908_m1	GUSB	Endogenous Control
Hs99999909_m1	HPRT1	Endogenous Control
Hs99999910_m1	TBP	Endogenous Control
Hs99999911_m1	TFRC	Endogenous Control

Table S3: Cell cycle distribution in Scr-siRNA and PDE4D-siRNA HCC cells.

HepG2					
**Phase**	**Mean**		**SD**		***P vs Scr***
	Scr-siRNA	*PDE4D-siRNA*	*Scr-siRNA*	*PDE4D-siRNA*	*P*
G0/G1	75.86	78.13	0.53	1.13	0.03
S	14.38	13.17	0.19	0.58	0.02
G2/M	9.38	8.29	0.43	1.10	0.18
**Hep3B**					
**Phase**	**Mean**		**SD**		***P vs Scr***
	Scr-siRNA	*PDE4D-siRNA*	*Scr-siRNA*	*PDE4D-siRNA*	
G0/G1	74.78233	72.49	2.79	2.39	0.03
S	18.75202	16.31	3.02	2.93	0.04
G2/M	5.740084	10.17	0.92	5.63	0.98
**Huh7**					
**Phase**	**Mean**		**SD**		***P vs Scr***
	Scr-siRNA	*PDE4D-siRNA*	*Scr-siRNA*	*PDE4D-siRNA*	
G0/G1	71.88	75.00	1.48	0.24	0.02
S	17.59	13.22	1.87	0.47	0.01
G2/M	10.08	11.39	2.56	0.27	0.42

Table S4: Cell cycle distribution in Vehicle-treated and Gebr-7b-treated HCC cells.

HepG2					
**Phase**	**Mean**		**SD**		***P vs Vehicle***
	Vehicle	*Gebr-7b*	*Vehicle*	*Gebr-7b*	
G0/G1	61.86	67.58	1.35	0.36	0.002
S	21.84	17.37	1.09	1.30	0.01
G2/M	16.28	14.47	0.31	0.98	0.10
**Huh7**					
**Phase**	**Mean**		**SD**		***P vs Vehicle***
	Vehicle	*Gebr-7b*	*Vehicle*	*Gebr-7b*	
G0/G1	54.00	59.77	3.48	7.90	0.035
S	34.67	30.08	2.59	6.12	0.005
G2/M	11.32	10.14	0.99	2.51	0.74

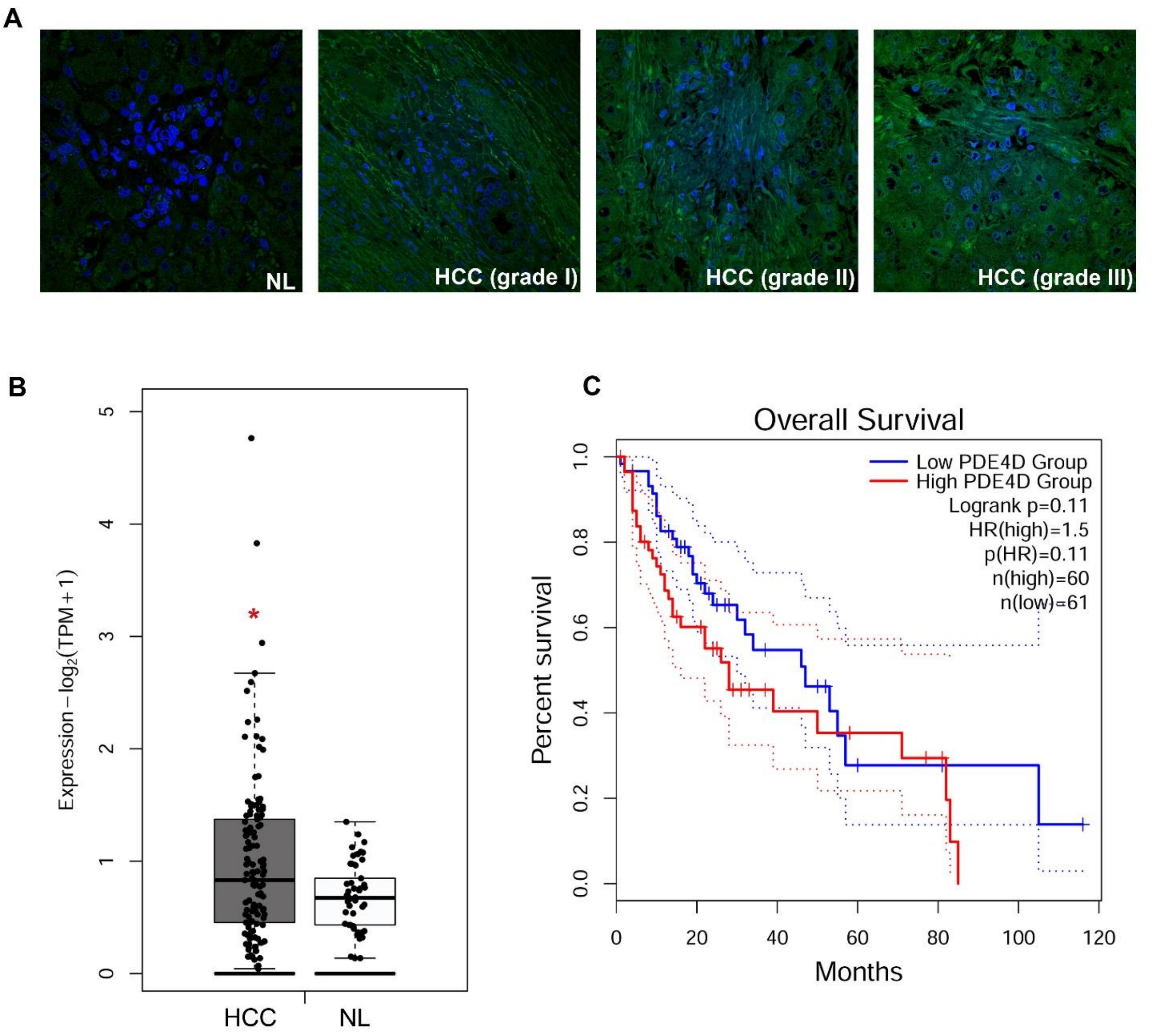

Figure S1. PDE4D expression levels in HCC samples compared to normal liver samples (NL). A) Representative immunofluorescence of PDE4D (green) in HCC tissues compared to control NL samples. Nuclei counterstaining with DAPI (blue). **B**) Box plot of PDE4D gene expression in HCC tissues compared to control NL samples from GEPIA database. Anova test. *P<0.05. **C**) Survival curve of differential PDE4D expression analysed by GEPIA database.

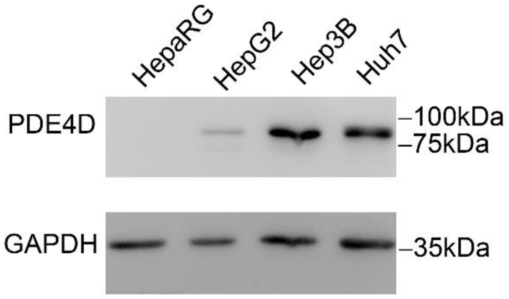

Figure S2. PDE4D protein expression in HCC cell lines. Representative immunoblotting of PDE4D measured in HepaRG, HepG2, Hep3B, and Huh7 cells.

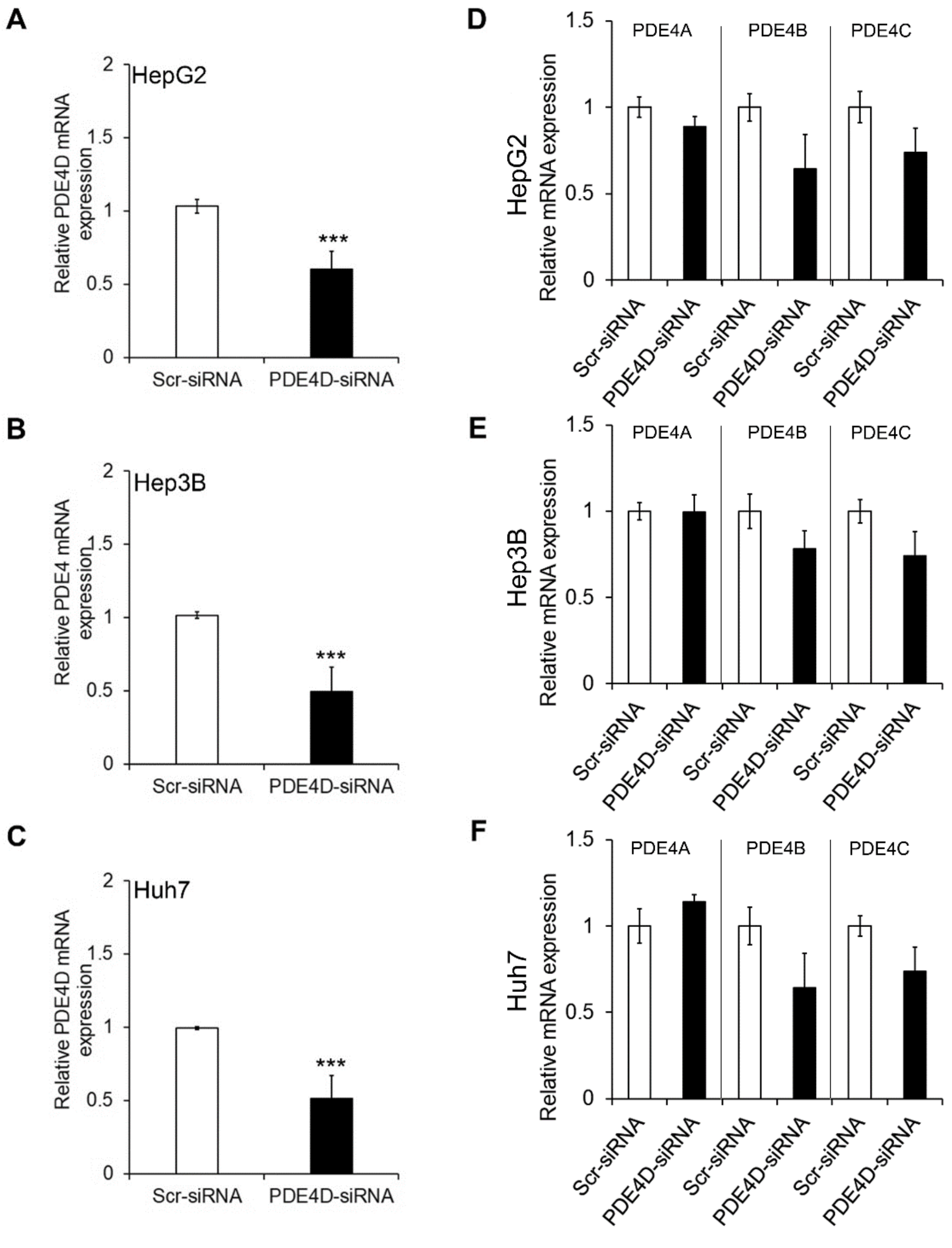

Figure S3. The effect of PDE4D silencing on the expression of PDE4D, PDE4A, PDE4B, and PDE4C mRNA in HCC cells. qRT-PCR analysis of mRNA levels of PDE4D in HepG2 (**A**), Hep3B (**B**), and Huh7 (**C**) cells 48 hours after transient silencing. qRT-PCR analysis of mRNA levels of PDE4A, PDE4B and PDE4D in HepG2 (**D**), Hep3B (E), and Huh7 (F) cells 48 hours after transient silencing. Data are the mean ± standard deviation (SD) of at least two independent experiments. Student t test. ***P<0.001 versus Scr-siRNA.

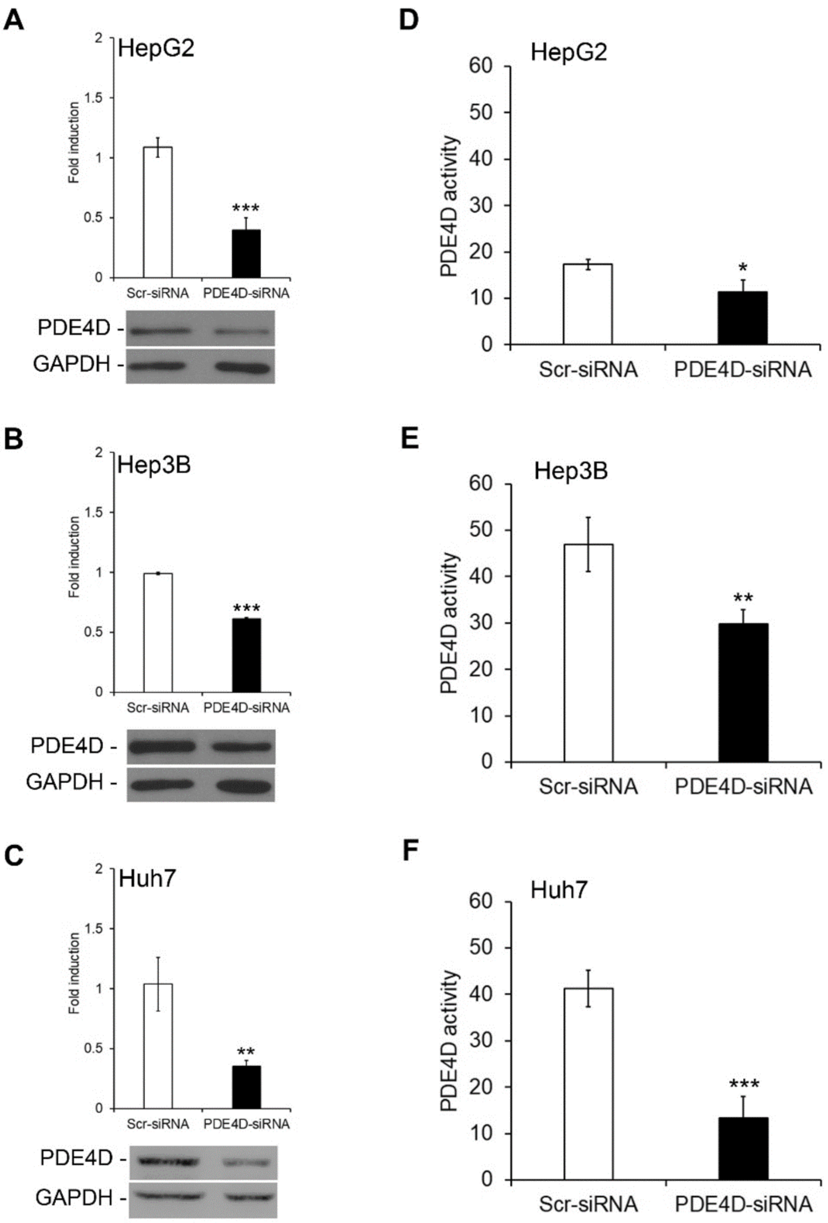

Figure S4. The effect of PDE4D silencing on its protein expression and activity in HCC cells. Quantitative densitometry of PDE4D protein normalized to GAPDH in HepG2 (**A**), Hep3B (**B**), and Huh7 (**C**) cells 48 hours after transient silencing. PDE4D activity in HepG2 (**D**), Hep3B (**E**), and Huh7 (**F**) cells 48 hours after transient silencing. Data are the mean ± standard deviation (SD) of three independent experiments. Student t test. *P<0.05; **P<0.01; ***P<0.001 versus Scr-siRNA.

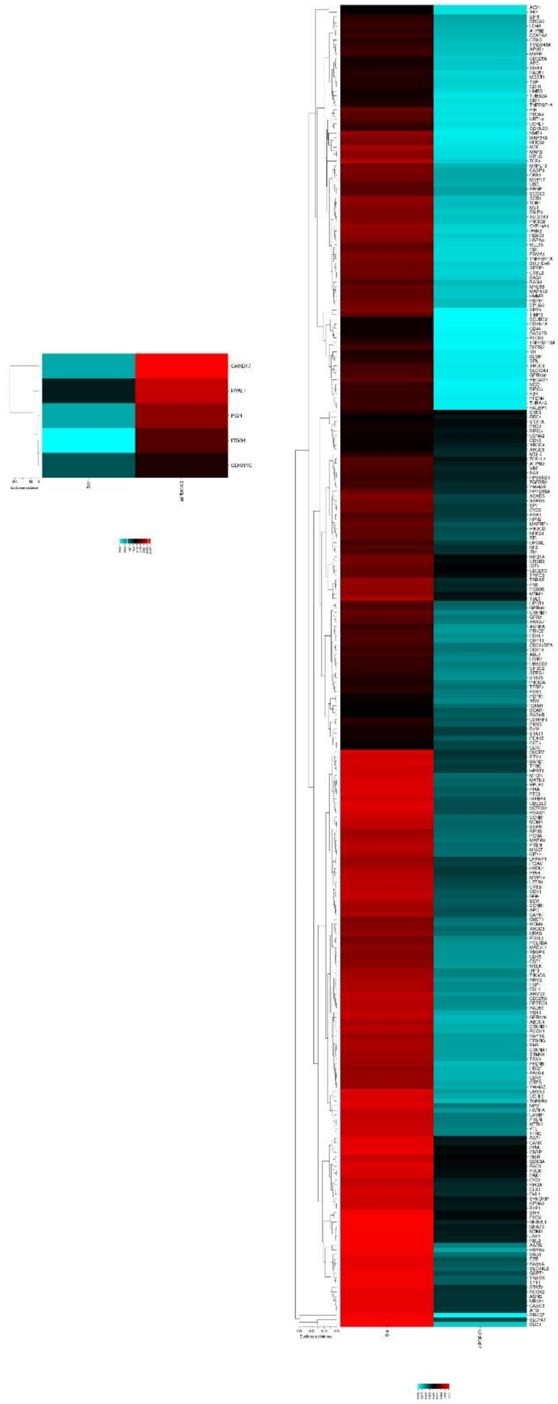

Figure S5. Gene expression analysis in Huh7 cells after silencing of PDE4D gene. Heatmap representation of cancer-related genes analysed by TaqMan OpenArray that were up-regulated (left panel) or down-regulated (right panel) in Huh7 cells silenced or not silenced for PDE4D. This image was generated using online tools provided by CIMminer (http://discover.nci.nih.gov/cimminer/home.do).

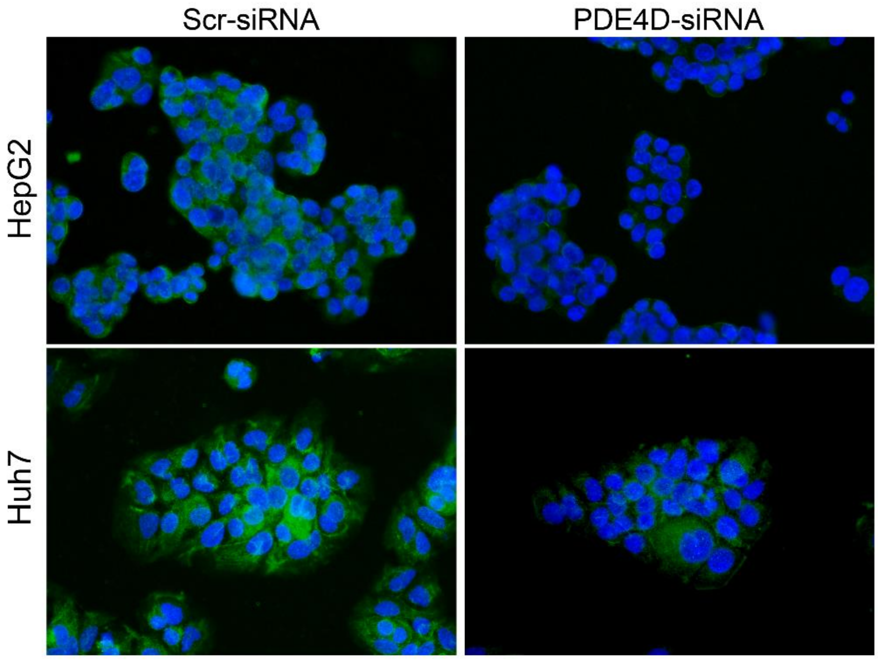

Figure S6: Expression of IGF2 protein upon PDE4D silencing in HCC cells. Representative immunofluorescence images of IGF2 protein (green). Nuclei were counterstained with DAPI (blue). Magnification 40X.

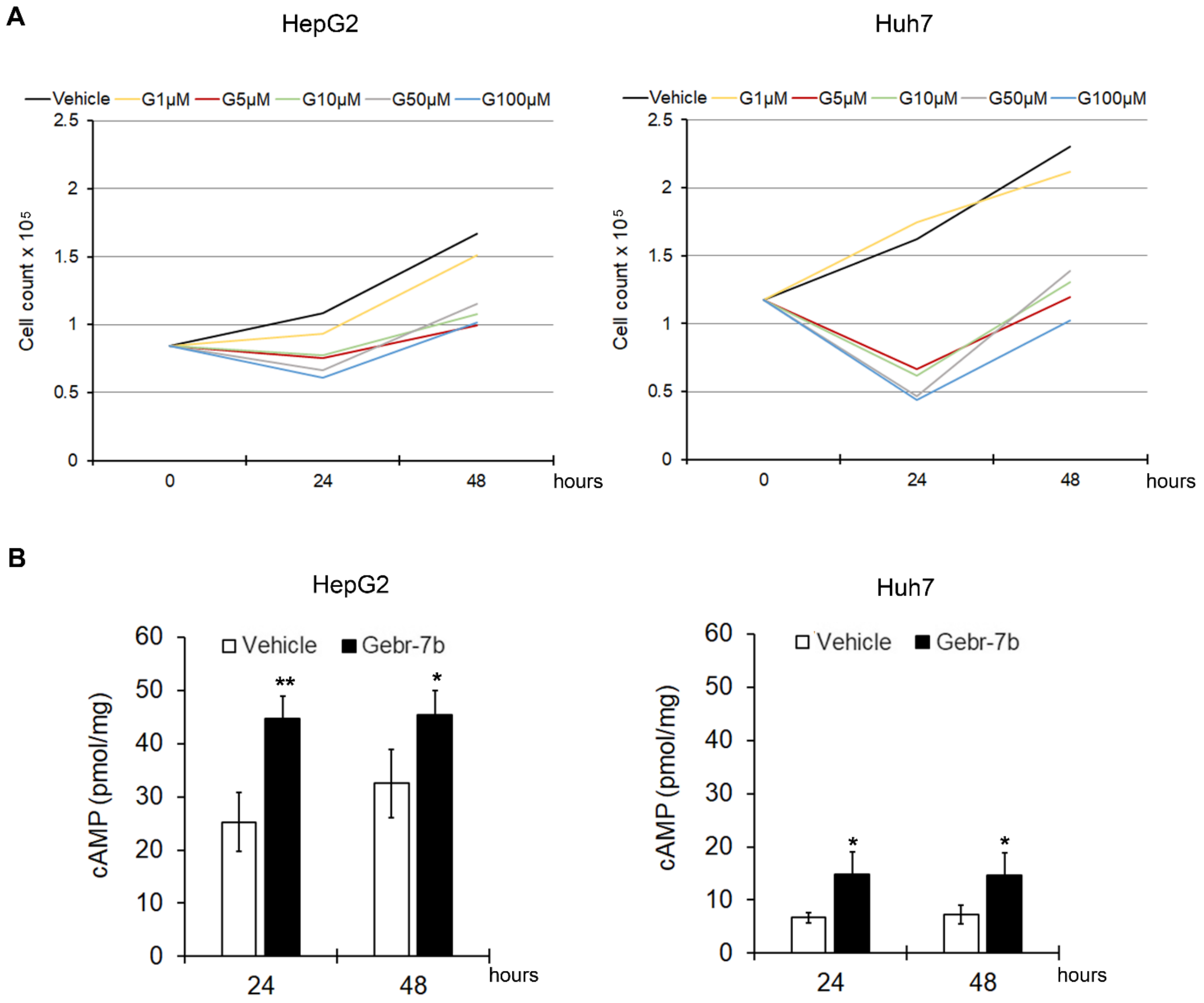

Figure S7. Evaluation of the effects of Gebr-7b on HCC cell growth and PDE4D activity. A) Growth curves at 24 and 48 hours of HepG2 and Huh7 cells treated or not treated with different amounts of Gebr-7b. **B)** cAMP levels in HepG2 and Huh7 cells treated or not treated with 5µM Gebr-7b at 24 and 48 hours. Data are the mean ± SD of three independent experiments. Student t test. **P*<0.05; ***P*<0.01 versus vehicle.
